# Stable, flexible, common, and distinct behaviors support rule-based and information-integration category learning

**DOI:** 10.1038/s41539-023-00163-0

**Published:** 2023-05-13

**Authors:** Casey L. Roark, Bharath Chandrasekaran

**Affiliations:** 1 Department of Communication Science & Disorders,University of Pittsburgh, Pittsburgh, PA USA; 2 Center for the Neural Basis of Cognition, Pittsburgh, PA USA

**Keywords:** Human behaviour, Cognitive neuroscience

## Abstract

The ability to organize variable sensory signals into discrete categories is a fundamental process in human cognition thought to underlie many real-world learning problems. Decades of research suggests that two learning systems may support category learning and that categories with different distributional structures (rule-based, information-integration) optimally rely on different learning systems. However, it remains unclear how the same individual learns these different categories and whether the behaviors that support learning success are common or distinct across different categories. In two experiments, we investigate learning and develop a taxonomy of learning behaviors to investigate which behaviors are stable or flexible as the same individual learns rule-based and information-integration categories and which behaviors are common or distinct to learning success for these different types of categories. We found that some learning behaviors are stable in an individual across category learning tasks (learning success, strategy consistency), while others are flexibly task-modulated (learning speed, strategy, stability). Further, success in rule-based and information-integration category learning was supported by both common (faster learning speeds, higher working memory ability) and distinct factors (learning strategies, strategy consistency). Overall, these results demonstrate that even with highly similar categories and identical training tasks, individuals dynamically adjust some behaviors to fit the task and success in learning different kinds of categories is supported by both common and distinct factors. These results illustrate a need for theoretical perspectives of category learning to include nuances of behavior at the level of an individual learner.

## Introduction

Across a variety of problems in perception and cognition, there is substantial variability across individuals. Individuals vary widely in how well they can learn arbitrary perceptual categories^1,2^, solve complex logic problems^3^, or learn a second language^4–6^. Across such a variety of tasks, some individuals learn quickly while others struggle to learn at all. Here, we investigate what underlies this variability and what behaviors during learning are associated with successful learning. Particularly, we focus on learning of two types of artificial categories that have been proposed to be optimally learned by separable cognitive and neural learning systems: rule-based (RB) and information-integration (II) categories^7^.

**Fig. 1:**
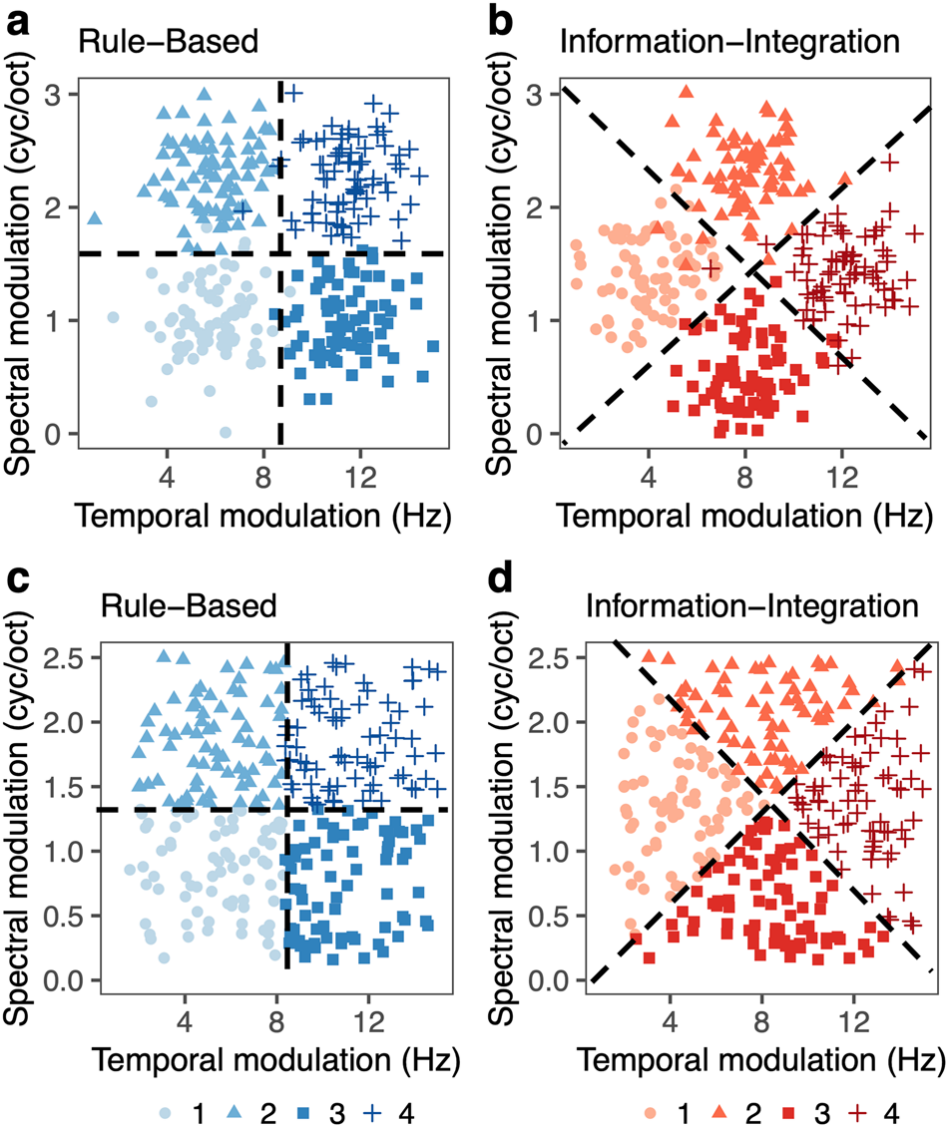
Category Distributions. Distributions for Experiment 1 (**a**, **b**) and Experiment 2 (**c**, **d**). **a**, **c** Rule-Based and **b**, **d** Information-Integration category structures varying in temporal modulation (Hz) and spectral modulation (cyc/oct). Dashed lines reflect optimal boundaries separating the categories that are either orthogonal (rule-based) or non-orthogonal (information-integration) to the underlying dimensions defining the stimuli.

RB and II categories are primarily distinguished from one another based on their distributional structure^7–9^. RB categories can be separated with decision boundaries that are orthogonal to the dimensions (Fig. 1a, c), whereas II categories can be separated with boundaries that are non-orthogonal to the dimensions (Fig. 1b, d). As a result of these differences in distributional structure, the requirements for optimal learning are somewhat different. Learning RB categories involves selective attention to the individual stimulus dimensions to create and find rules that separate the categories into quadrants (e.g., Category 1 is low on both temporal and spectral modulation). In contrast, learning II categories involves integration along the dimensions in a way that cannot be easily verbalized by the learner (e.g., Category 1 is low on temporal modulation except when it is not and then it has medium levels of spectral modulation compared to Categories 2 and 3). In this case, making decisions based on the similarity to other encountered stimuli may be a better overall strategy than creating a rule^10^. Rather than only applying to artificial experimental contexts, these types of categories may reflect the structure of real-world learning problems that are either distinguished by simple rules (e.g., differentiating tenor and bass singers by pitch frequency range) or more complex rules that are difficult to describe verbally (e.g., differentiating the sounds /b/ and /p/ by whether the vocal cords vibrate while producing the sound).

There are two opposing views about the nature of the mechanisms supporting learning RB and II category structures. Multiple systems theories (e.g., Competition between Verbal and Implicit Systems [COVIS] theory^11^) posit that separate learning systems are best suited for RB (explicit, hypothesis testing system) and II (implicit, procedural learning system) learning^9,11–26^. In contrast, single systems theories posit that a single system (e.g., exemplar, prototype models^27–31^) can account for both RB and II category learning and suggest that dissociations seemingly supporting multiple systems perspectives on learning have significant methodological or theoretical limitations^32–35^.

Regardless of whether the evidence supports or refutes a dissociation between RB and II category learning, much of the existing work on RB and II category learning has focused on the possible differentiation of these two learning systems using manipulations that influence group means. As a result, not much is understood about how the same individual learns RB and II categories and whether the behaviors that support learning are common or distinct across these types of categories. In the current work, we introduce a taxonomy of behaviors during category learning that enables a deeper investigation into learning and examine whether behaviors are stable or flexible in a learner across tasks and common or distinct to RB and II learning success.

Several prior studies have examined RB and II learning in the same individuals. However, these studies have not compared behavior across tasks and, instead, focused on between-subjects comparisons (musicians vs. non-musicians^36^; Parkinson’s Disease patients vs. older adults vs. younger adults^37^), examined RB and II learning in a task-switching context^38^, or focused on how individual differences in cognitive abilities relate to RB and II learning^39^. As a result, it is unclear how the same individual approaches and learns RB and II categories. One possibility is that an individual is highly stable in their behavior across different category learning tasks, while an alternative possibility is that an individual flexibly adjusts their behavior depending on the task context or their current state.

To understand what might be commonly or distinctly related to RB and II learning success, prior lines of work have focused on individual differences in working memory (WM) capacity. The role of WM in learning has been explored extensively^40–43^. The focus on WM capacity in RB and II category learning stems from the proposal in the COVIS theory that the explicit system selectively relies on WM capacity and prefrontal cortex, where the implicit system relies on striatal procedural learning mechanisms^11^. In line with COVIS theory’s proposal that optimal RB learning involves the explicit system, there is consistent evidence that WM relates to successful RB learning^39,44,45^. The empirical evidence for the role of WM in II learning is less clear. In line with COVIS theory’s proposal that WM may be minimally involved in optimal II learning^11^, some studies have found that higher WM has minimal or negative impact on II learning^46–49^. However, others have found that that higher WM is better for learning in general, regardless of what type of category is being learned^39,45,50–52^. Importantly, even when WM capacity has been found to be related to learning, the explanatory power of WM is usually moderate, leaving substantial unexplained variance in category learning ability^39,45,53^. There is still much to be understood about what supports learning beyond WM capacity.

An important component to understanding individual differences in learning is identifying variability in behavior during learning in addition to stable participant abilities like WM capacity. Many studies have demonstrated that even within the same task, participants vary in the strategies they use to learn^1,2,17,23,36,45,54–57^. Studies grounded in the COVIS theory typically rely on estimation of learning strategies using strategy modeling approaches^58–60^. Through this framework, models reveal how participants use the underlying stimulus dimensions to make decisions about category identity and whether learners rely on explicit or implicit learning mechanisms. However, there are meaningful criticisms about estimation of learning strategies through strategy modeling approaches and whether this actually reflects the involvement of different systems in learning^61,62^. As a result, there are theoretical reasons to move beyond learning strategy to investigate individual differences in learning behavior.

**Fig. 2:**
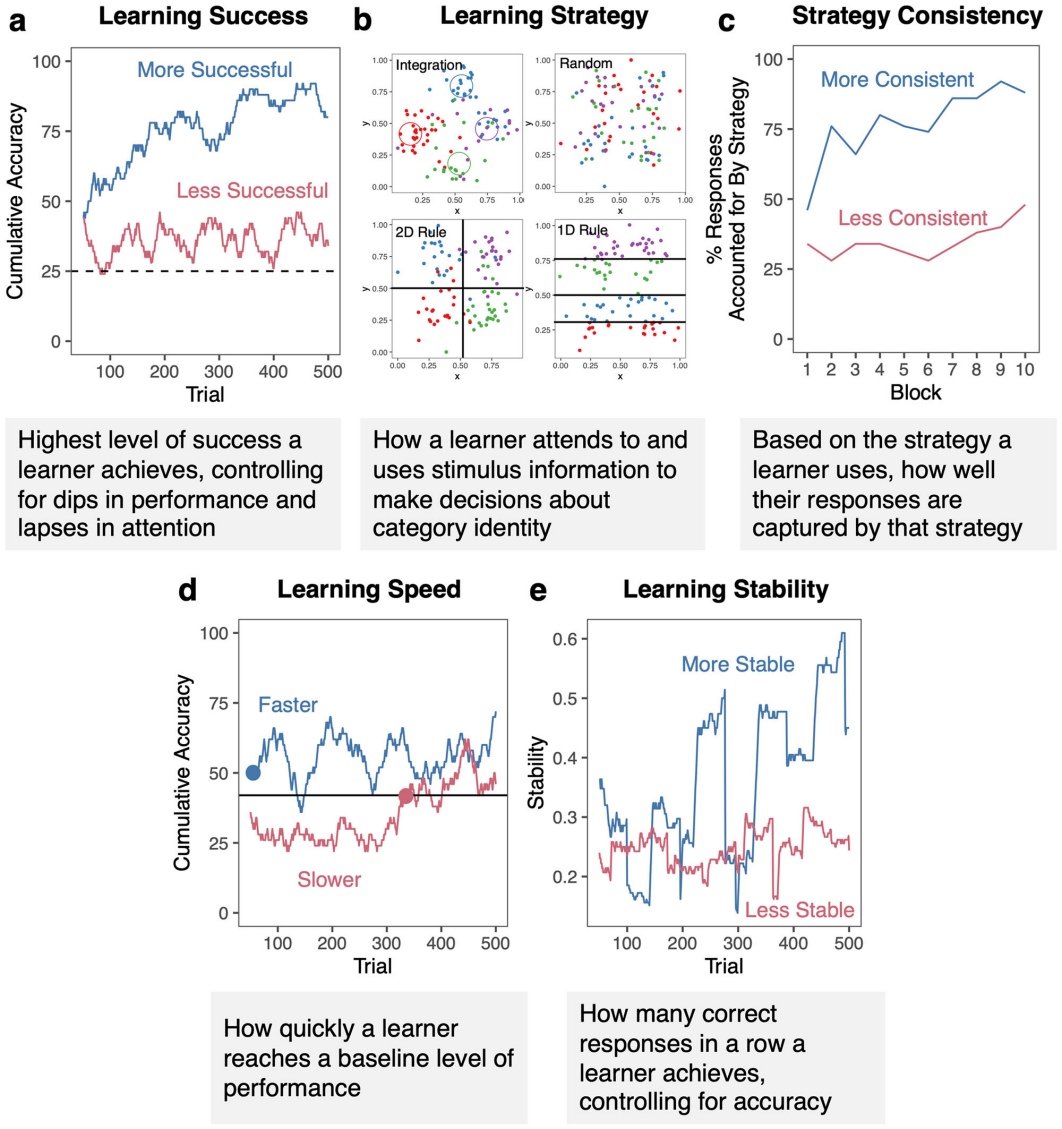
Taxonomy of Category Learning Behaviors. **a** Learning success with two representative learners who were more successful (92% maximum cumulative accuracy) and less successful (46% maximum cumulative accuracy). **b** Illustration of the four possible learning strategies: integration based on similarity, random guessing, two-dimensional (2D) or one-dimensional (1D) rules. **c** Strategy consistency for two representative learners who had more consistent (92% maximum consistency) or less consistent (46% maximum consistency) strategies. **d** Learning speed illustrated in two representative learners who had faster (0) or slower (282) speeds to reach the group median (black line). Note that their learning success was relatively similar (72% and 62% respectively). **e** Learning stability as the number of correct responses in a row divided by the total number of correct responses over a moving window of 50 trials with two representative learners who are more stable (0.61 maximum stability) or less stable (0.32 maximum stability). Note that their learning success was relatively similar (92% and 90% respectively).

With the goal of understanding individual differences in learning behavior across tasks and which behaviors are associated with RB and II learning, we present a taxonomy of behaviors during category learning (Fig. 2). To complement prior approaches, we examine how WM capacity and learning strategies are associated with learning success in RB and II tasks. Going beyond these prior approaches, we examine how an individual’s behavior and learning success across different tasks are related to the consistency of one’s learning strategy, learning speed, and learning stability.

We examine learning success – the highest level of success a learner achieves, controlling for dips in performance or lapses in attention. We examine learning strategy – how a learner uses underlying stimulus information to make decisions about category identity. We move beyond examining the type of strategies learners use to understand their strategy consistency – how consistently learners apply whichever type of strategy they use during learning. We examine learning speed – how quickly learners reach a baseline level of performance. Learning speed reflects the ability to quickly acquire workable, though likely suboptimal, rules to define the categories. Faster learning speeds may generally provide learners more ‘room to grow’ and improve their accuracy further. Alternatively, faster learning speeds may be unrelated to overall learning if participants stall in performance and do not continue to improve. Finally, we examine learning stability – streaks of correct responses while controlling for overall accuracy. Higher learning stability (e.g., longer streaks of correct responses) is generally thought to be reflective of learning, as prior studies have used streaks of correct responses as an overall measure of learning^46,63–65^. However, it is unclear if stability in performance is related to overall learning when controlling for number of overall correct responses.

This new taxonomy is particularly useful because we can provide in-depth descriptions of category learning behavior and make predictions about how these behaviors may be stable in an individual or flexible to a task context and commonly or distinctly related to success in RB and II tasks.

It is possible that these learning behaviors are stable in an individual across tasks, reflecting a stable trait or behavioral tendency. In highly controlled artificial category learning tasks, as we will examine here, the tasks are supervised learning tasks with identical feedback, goals, and stimulus dimensions. As a result, there are many reasons to expect that the same person will approach these highly similar tasks in similar ways – applying similar learning strategies, learning similarly quickly, and having similar stability in performance. In support of this prediction, there is some evidence that people tend to use similar simple rule strategies during learning regardless of what is optimal for the task^14,66^. Here, we will investigate if the *same person* applies similar strategies and learning behaviors during RB and II tasks with distinct learning demands.

Alternatively, these behaviors may be unrelated across tasks and, instead, an individual may modulate their behavior depending on the specific task context. This prediction is well-aligned with a dual systems perspective of learning and suggests that how an individual behaves during learning will be modulated based on what is optimal for learning (i.e., whether they are learning RB or II categories). That is, participants will adjust their strategy, how consistently they use their strategy and will differ in how quickly they learn and how stable their performance is depending on what is required of them in the task. Additionally, this prediction would also be well-aligned with a perspective that suggests a heavy influence of a learner’s specific motivational or attentional state within a specific task^67–70^. For example, a learner may be highly motivated and attentive one day and less so on another day.

Regardless of how these behaviors are related within an individual as they complete different tasks, there are reasons to expect that learning in RB and II tasks may be supported by common behaviors. The underlying task and goals are identical. If this supervised learning task, rather than the distributions of the categories, is a major driver of behavior, we would expect to see mostly common behaviors relating to success in RB and II tasks. Specifically, learners may find varying levels of success with different strategies and reach higher levels of success the more consistently the apply a strategy, the faster they reach a baseline level of performance, and the more stable their learning is regardless of what kind of category they are learning.

In contrast, stemming from the dual systems perspective of COVIS, there are reasons to expect that RB and II learning may be supported by mostly distinct behaviors. If RB learning relies on the explicit system, RB learning may be uniquely supported by consistent use of explicit-system strategies, faster learning speeds, earlier learning stability, and higher WM capacity. Explicit-system strategies are optimal for RB learning^7,11^, faster learning speeds may reflect use of explicit-system rules^63,71^, earlier learning stability should be achieved if learners quickly find useful explicit-system rules^63,72^, and higher WM capacity has been consistently linked to better RB learning^39,45,54,73^. If II learning relies on the implicit system, II learning may be uniquely supported by consistent use of implicit-system strategies and may be unrelated to learning speed, learning stability, or working memory capacity. Implicit-system strategies are optimal for II learning^11^, optimal II learning takes longer as it is reliant on slowly building up stimulus-response associations^7,74^, and higher WM capacity may not be beneficial for II learning^46–49^.

We first test the taxonomy of learning behaviors in Experiment 1 and then further test the generalizability and replicability of the taxonomy in Experiment 2. As the paradigms and predictions are identical across experiments, we present results together and highlight when results do or do not replicate across experiments. In both experiments, we examine RB and II auditory category learning in the same individuals in two separate sessions. We use the taxonomy of behaviors during category learning to assess the stability and flexibility of behaviors in the same individual across tasks and to identify the common and distinct behaviors supporting RB and II learning.

## Results

### Summary of experiments

In Experiment 1, 86 (36 F, *M* = 25.4 years, SD = 5.04 years) participants learned both RB and II categories (Fig. 1a, b) across two sessions separated by at least a week. We first examine how an individual’s behavior is related across RB and II category learning to understand within-subject variability in learning. We then examine how these behavioral metrics relate to success in a way that is common or distinct across the two tasks. Finally, we assess the possible contribution of order effects by examining carryover effects in both accuracy and strategies. We did not exclude any participants based on performance.

Experiment 2 served as a replication and extension. The experimental design differed in three ways: (1) the same stimulus distributions were used for RB and II categories to eliminate potential concerns about biases in results stemming from differences in category distributions^75^, (2) we trained participants for an additional 100 trials (600 trials total) to give participants a better chance to learn, and (3) we directly asked participants after each task how they determined which sounds belonged to which categories. We did not analyze the response data here but provide the data on OSF for the benefit of other interested researchers. In Experiment 2, 93 (38 F, *M* = 30.1 years, SD = 4.66 years) participants learned both RB and II categories (Fig. 1c, d) across two sessions separated by at least a week. We examine the same measures for Experiment 2 as Experiment 1.

### Which behaviors are flexible or stable in across tasks?

**Fig. 3:**
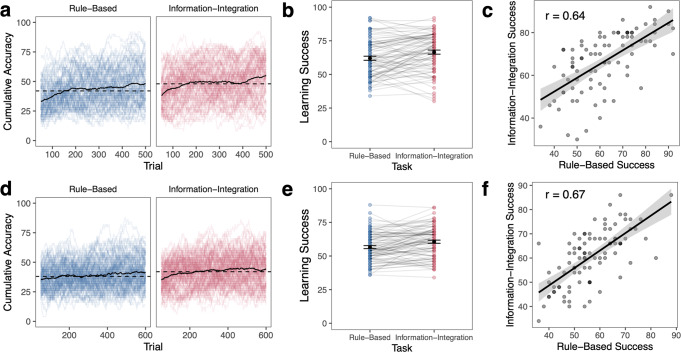
Learning success across tasks. For Experiment 1 (**a**–**c**) and Experiment 2 (**d**–**f**), **a**, **d** Cumulative accuracy over a moving window of 50 trials, first plotted at trial 50. The solid black line reflects mean over all subjects at each trial timepoint. Colored lines reflect variability across individuals. The dashed black line reflects median level of performance within the task (Experiment 1: II: 48%, RB: 42%; Experiment 2: II: 42%, RB: 38%). **b**, **e** Learning success as maximum cumulative accuracy across all timepoints for individual subjects. Error bars reflect s.e.m. **c**, **f** Correlation between maximum accuracy in the RB and II tasks. Error ribbon reflects s.e.m.

**Fig. 4:**
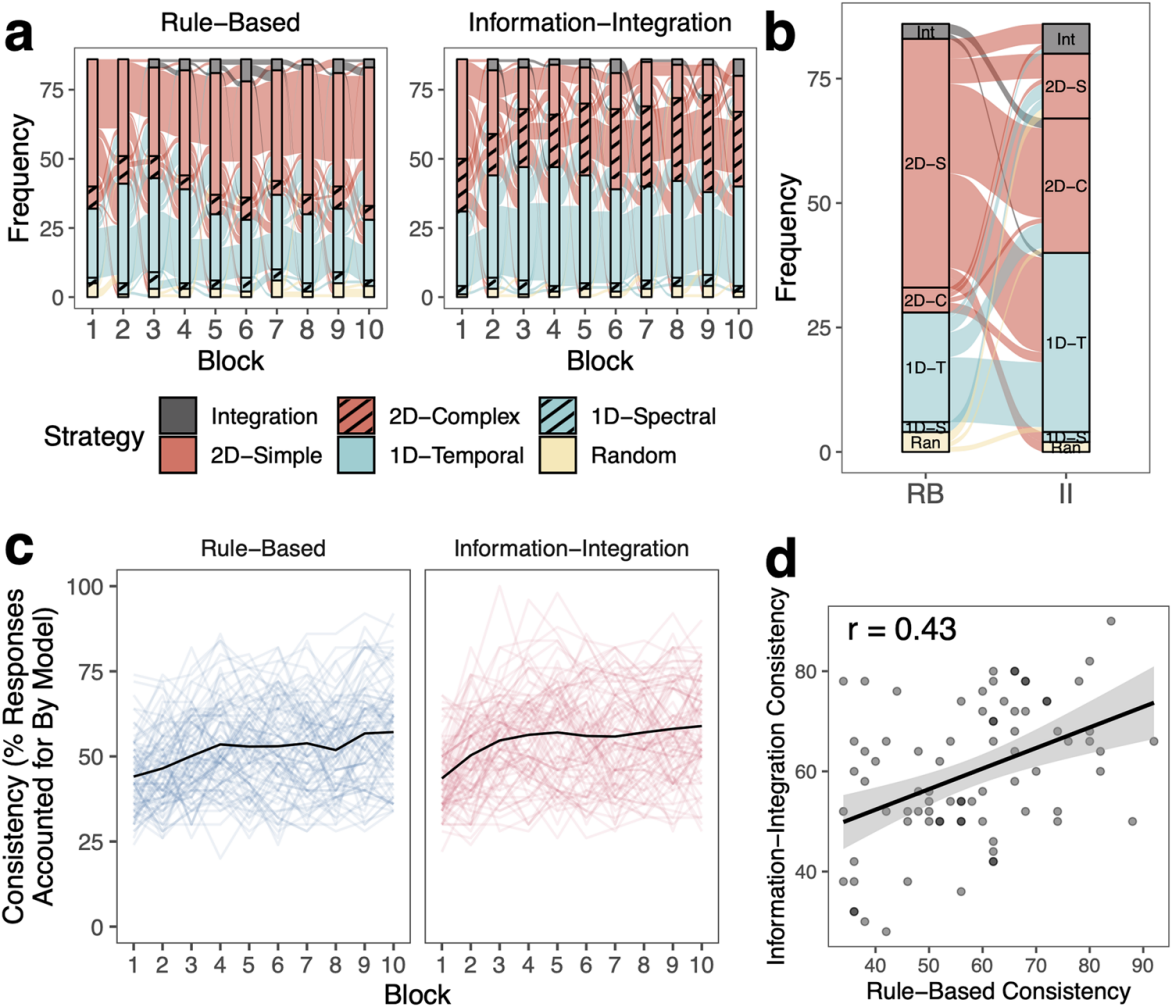
Learning strategies across tasks in Experiment 1. For Experiment 1 (**a**) Strategies across blocks. Colors reflect different classes of strategies, which are binned by frequency. Connections between blocks show how strategies changed across blocks. **b** Alluvial plot showing the strategies the same participants used in the final block of the RB and II tasks. **c** Consistency of strategy application measured as the percent of a participant’s responses that were accounted for by the best-fitting model/strategy. 100% consistency would reflect that the participant clearly applied this strategy with no exceptions in any trials. 25% consistency would reflect a relatively poor fit of the model to the participant’s data. Consistency is only measured for non-random strategies. Mean is shown as the black line and individual data is shown in colored lines. **d** Correlation between final-block consistency in the RB and II tasks. Error ribbon reflects s.e.m.

**Fig. 5:**
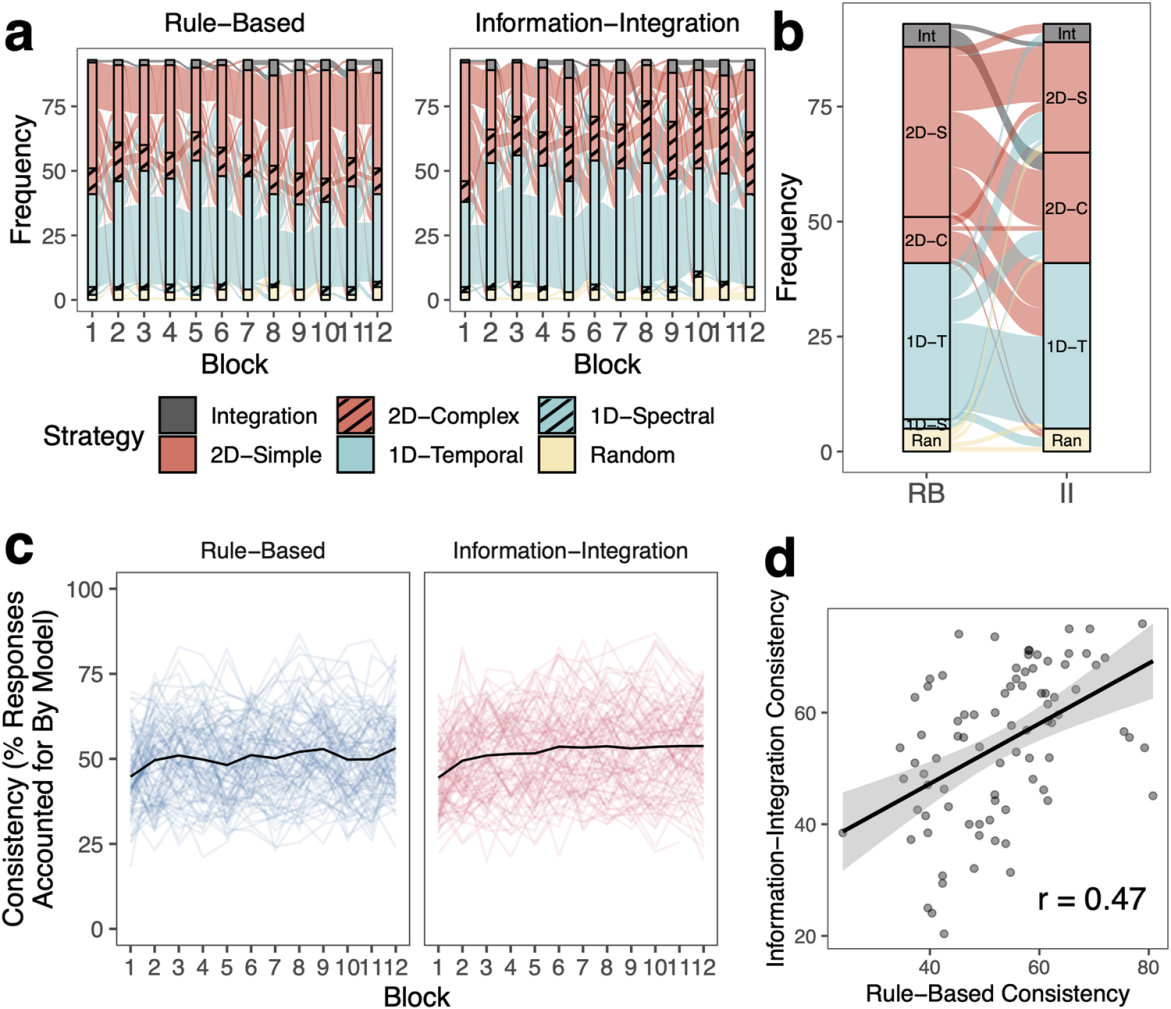
Learning strategies across tasks in Experiment 2. For Experiment 2 (**a**) Strategies across blocks. Colors reflect different classes of strategies, which are binned by frequency. Connections between blocks show how strategies changed across blocks. **b** Alluvial plot showing the strategies the same participants used in the final block of the RB and II tasks. **c** Consistency of strategy application measured as the percent of a participant’s responses that were accounted for by the best-fitting model/strategy. 100% consistency would reflect that the participant clearly applied this strategy with no exceptions in any trials. 25% consistency would reflect a relatively poor fit of the model to the participant’s data. Consistency is only measured for non-random strategies. Mean is shown as the black line and individual data is shown in colored lines. **d** Correlation between final-block consistency in the RB and II tasks. Error ribbon reflects s.e.m.

**Fig. 6:**
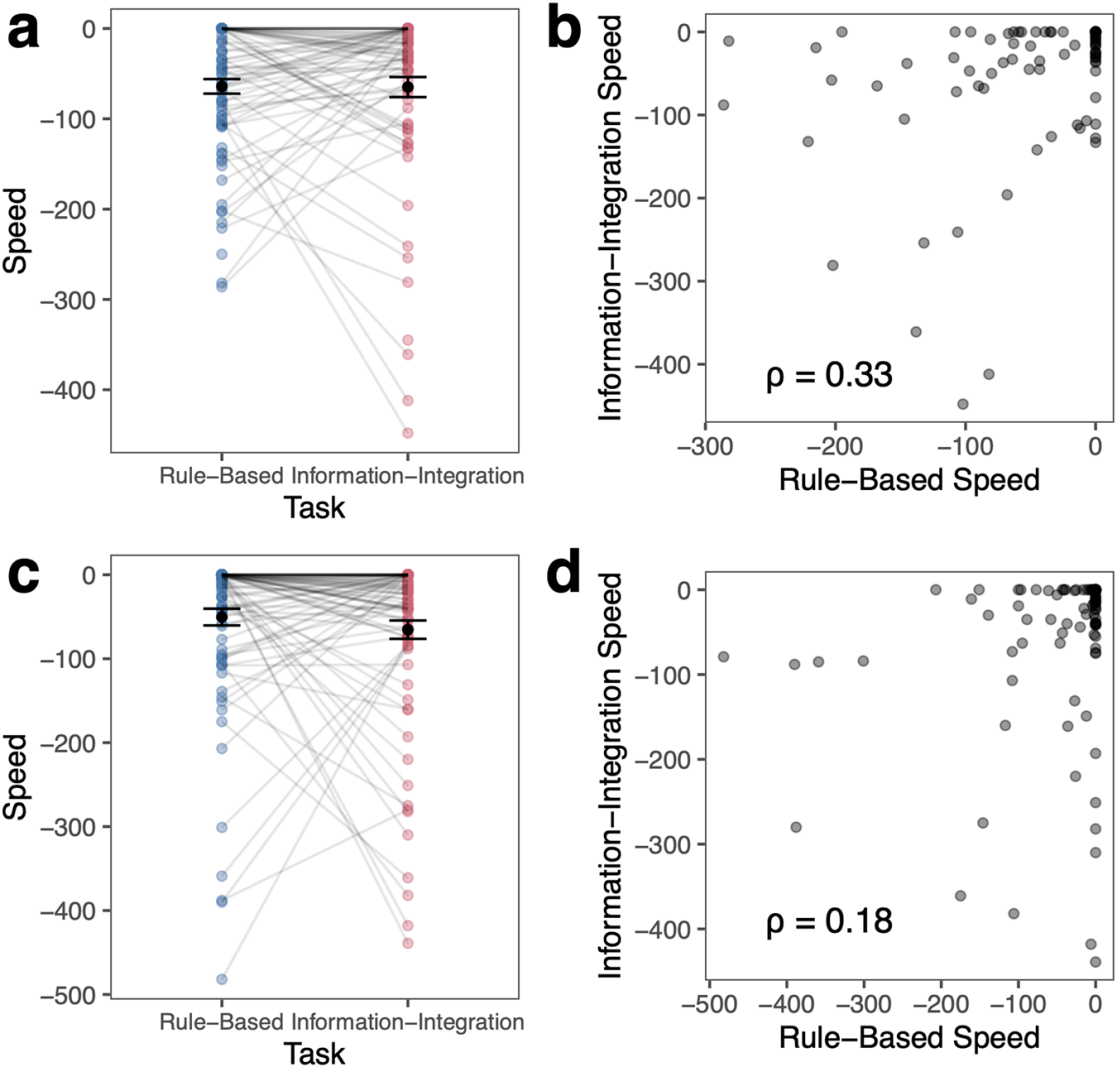
Learning speed across tasks. For Experiment 1 (**a**, **b**) and Experiment 2 (**c**, **d**), **a**, **c** learning speed calculated as the point in the moving window of 50 trials where the learner first met or exceeded the group median for that task over all timepoints (Experiment 1: II: 48%, RB: 42%; Experiment 2: II: 42%, RB: 38%). Scores are inverted so higher values reflect faster learning speeds. Zero reflects that they reached this level of performance within the first 50 trials, with additional decreases in speed reflecting the additional number of trials it took for the learner to reach the median level of performance. Ten participants in Experiment 1 and five participants in Experiment 2 were excluded because they never reached the median in at least one task. Error bars reflect s.e.m. **b**, **d** Correlation between learning speed in the RB and II tasks, with spearman’s *ρ* reported.

**Fig. 7:**
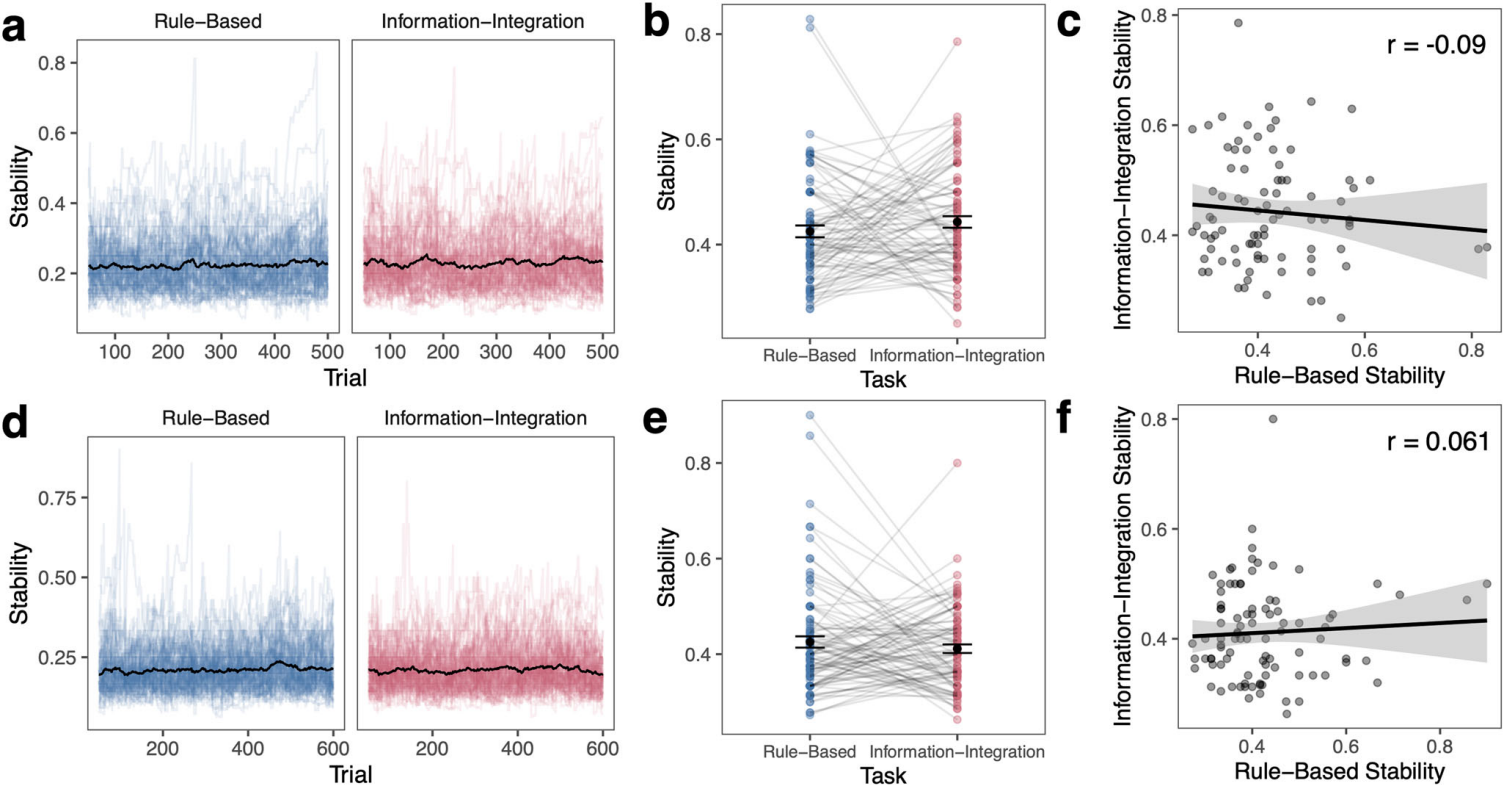
Learning stability across tasks. For Experiment 1 (**a**–**c**) and Experiment 2 (**d**–**f**), **a**, **d** learning stability calculated as the longest streak of correct responses over the previous 50 trials, normalized by total number of correct responses over those 50 trials. Mean is shown as a black line and individual data is shown as colored lines. **b**, **e** Learning stability as maximum stability in each task for each participant and the group mean. Error bars reflect s.e.m. **c**, **f** Correlation between learning stability in the RB and II tasks. Error ribbon reflects s.e.m.

To understand how behaviors are related across RB and II tasks in the same individual, we compared overall learning success (Fig. 3), learning strategies and the consistency of the application of those strategies (Figs. 4 and 5), learning speed (Fig. 6), and learning stability (Fig. 7). These measures provide information of both how well participants learn and how they learn in these two similar tasks with distinct requirements.

#### Learning success

To understand how well participants learned in the RB and II tasks, we computed their cumulative accuracy over a moving window of 50 trials (Fig. 3a, d). This provides information about how performance evolves over time and demonstrates that there is wide variability across individuals. We computed participants’ overall learning success as the maximum value of this cumulative accuracy. In Experiment 1, participants tended to have higher learning success in the II task (*M* = 67%) compared to the RB task (*M* = 62%, *t*(85) = 3.55, *p* = 0.00063, *d* = 0.38, 95% CI [2.00, 7.11]; Fig. 3b). Learning success was strongly correlated across the RB and II tasks (*r*(84) = 0.64, 95% CI [0.50, 0.75], *p* < 0.001; Fig. 3c). This pattern directly replicated in Experiment 2 – performance was significantly better in the II task (*M* = 61%) compared to the RB task (*M* = 57%, *t*(92) = 4.37, *p* = 0.000033, *d* = 0.45, 95% CI [2.19, 5.85]; Fig. 3e) and success was strongly correlated across tasks (*r*(92) = 0.67, 95% CI [0.55, 0.77], *p* < 0.001; Fig. 3f).

#### Consistency of learning strategies

Using strategy models including decision bound models^59,60^, we examined participants’ learning strategies across the 10 blocks of 50 trials in Experiment 1 (Fig. 4a) and 12 blocks of 50 trials in Experiment 2 (Fig. 5a). Specifically, we identified whether learners used one-dimensional (1D) rules (1D-Temporal or 1D-Spectral) or two-dimensional (2D) rules (2D-Simple or 2D-Complex), hypothesized to be linked to the explicit learning system, an integration strategy combining the dimensions in a way that is difficult for learners to describe verbally and hypothesized to be linked to the implicit learning system (Integration), or a random guessing strategy (Random).

In both experiments, for both RB and II tasks, participants primarily used 2D and 1D strategies to separate the stimuli into categories. The most common strategy for the RB task was the optimal 2D-Simple rule strategy, using simple conjunctive rules along both dimensions. The 2D-Simple rule strategy is optimal for RB categories in that the best 2D strategy can yield the highest accuracy in this task (Table 1; Experiment 1: 97%; Experiment 2: 100% vs. 25% chance). In contrast, the Integration strategy is optimal for II categories (Experiment 1: 95%; Experiment 2: 100%). Even if a participant used a suboptimal strategy during learning, the optimal strategies are always 2D-Simple rules for the RB task and Integration for the II task because the feedback participants receive is aligned with these optimal strategies. The most common strategy for the II task was the 1D rule strategy, particularly along the temporal modulation dimension (1D-Temporal). While this strategy is not optimal, the best 1D rule strategy can still yield moderate accuracy in this task (Experiment 1: 65%; Experiment 2: 55%).

**Table 1:** Highest possible accuracies of different strategies.

	1D-temporal rule (%)	1D-spectral rule (%)	2D-simple rule (%)	2D-complex rule (%)	Integration (%)	Random (%)
Experiment 1						
Rule-based	49	49	97	50	97	21–29^a^
Information-integration	65	65	50	89	95	21–29^a^
Experiment 2						
Rule-based	52	54	100	54	100	21–29^a^
Information-integration	55	58	52	76	100	21–29^a^

^a^95% cumulative probability over 300 trials with probability of correct response at 0.25, if a person was randomly guessing, they could expect to be correct on 21–29% of trials with 95% cumulative probability based on binomial probability distribution.

To understand how learning strategies were related across RB and II tasks in the same individual, we examined the types of strategies participants used in the final blocks of the two tasks (Figs. 4b and 5b). In both experiments, we found that strategies were generally unrelated across the two tasks, whether considering the specific strategy subclass (e.g., Integration, 2D-Simple, 2D-Complex, 1D-Temporal, 1D-Spectral, Random; Experiment 1: 78% different; Experiment 2: 65% different) or more general strategy class (e.g., Integration, 2D, 1D, Random; Experiment 1: 56% different; Experiment 2: 49% different).

However, some types of strategy combinations were more common than others. Among all participants in Experiment 1, 22% used a 2D-Simple strategy in the RB task and 1D-Temporal strategy in the II task (11% in Experiment 2), 21% used a 2D-Simple strategy in the RB task and a 2D-Complex strategy in the II task (13% in Experiment 2), and 15% used a 1D-Temporal strategy in both tasks (20% in Experiment 2). Another common strategy in Experiment 2 was using a 2D-Simple strategy in both tasks (13% in Experiment 2 and 6% in Experiment 1). Other strategy combinations accounted for between 0% and 6% of participants in both experiments. In all, we found that participants tended to use different strategies across RB and II tasks, adjusting their behavior based on the feedback they receive.

We were also interested in understanding how consistently participants applied whatever learning strategy they used. We calculated their strategy consistency as the proportion of their responses in a block that were accounted for by the best-fitting strategy. Perfect consistency (100%) would indicate that they applied that strategy consistently regardless of whether the strategy was optimal or suboptimal. In contrast, a consistency of 25% would mean that they inconsistently applied their strategy, and the best-fitting strategy was not a good fit to the data (i.e., capturing responses around chance levels: 25% for four categories).

Overall, we found that participants varied in the consistency of their strategy application, but generally became more consistent over the course of training (Figs. 4c and 5c). We compared how consistently the same participant applied their strategies across RB and II tasks by examining the correlation between their final-block consistency across tasks (Figs. 4d and 5d). Note that consistency cannot be calculated for those using random strategies, so participants using a random strategy in either task were not included in the analyses (Experiment 1: *N* = 6; Experiment 2: *N* = 10). In Experiment 1, there were no significant differences in strategy consistency across RB and II tasks (*t*(79) = 1.15, *p* = 0.25, *d* = 0.13, 95% CI [−1.43, 5.38]) and consistency was moderately correlated across the in RB and II tasks (*r*(78) = 0.43, 95% CI [0.23, 0.59], *p* < 0.001). This pattern replicated in Experiment 2, with no significant differences in strategy consistency across tasks (*t*(83) = 0.84, *p* = 0.40, *d* = 0.092, 95% CI [−1.65, 4.06]) and consistency was moderately correlated across tasks (*r*(82) = 0.47, 95% CI [0.28, 0.62], *p* < 0.001). Even when participants used different types of strategies across RB and II tasks, the consistency with which they applied whichever strategy they used was positively correlated across tasks. See supplementary materials for evidence that our results do not depend on using the same data to estimate strategy and calculate strategy consistency.

#### Learning speed

We next examined how quickly participants learned to distinguish the categories and reach the group median level of performance (Fig. 6a, c). We calculated learning speed as the point in the moving window of 50 trials where the learner first met or exceeded the group median performance over the entire task. We normalized learning speed by the group median for the two tasks in both experiments separately (Experiment 1: RB: *Mdn* = 42%, II: *Mdn* = 48%; Experiment 2: RB: *Mdn* = 38%, II: *Mdn* = 42%) to control for inherent differences in difficulty across the tasks and experiments. We then inverted the scores so higher values reflect faster learning speeds. A learning speed of zero indicates that the learner reached the median level of performance within the first 50 trials. Additional decreases in speed reflect the additional number of trials that it took for the learner to reach the median level of performance. Ten participants in Experiment 1 and six participants in Experiment 2 who never reached the median level of performance were excluded from the analyses (Experiment 1: both tasks, *N* = 2, RB task only, *N* = 2, II task only, *N* = 6; Experiment 2: both tasks, *N* = 1, RB task only: *N* = 1, II task only: *N* = 3). Many learners had the fastest possible learning speed (0) in at least one of the tasks. Due to the nature of the distribution of learning speeds across tasks, we calculated the Spearman’s rank correlation to understand the relationship of speed across tasks.

On average, in Experiment 1, participants reached the median performance after 59 trials in the RB task and 62 trials in the II task. Learning speed was not significantly different in the RB and II tasks (*t*(75) = 0.23, *p* = 0.82, *d* = 0.026, 95% CI: [−20.2, 25.5]) and learning speed was weakly monotonically related across the two tasks (spearman’s *ρ* = 0.33, *p* = 0.0032; Fig. 6b). This pattern nearly replicated in Experiment 2. Participants reached the median performance after 52 trials in the RB task and 65 trials in the II task on average. Learning speed was not significantly different across tasks (*t*(87) = 0.98, *p* = 0.33, *d* = 0.10, 95% CI [−13.7, 40.2]). In contrast to Experiment 1, learning speed was not significantly monotonically related across the two tasks (spearman’s *ρ* = 0.18. *p* = 0.086; Fig. 6d).

#### Learning stability

Finally, we calculated learning stability in a moving window of 50 trials as the longest streak of correct responses over the previous 50 trials, normalized by the total number of correct responses over those 50 trials (Fig. 7a, d). Higher values indicate higher performance stability. For example, if two participants responded correctly on 40 of 50 trials, their accuracies would both be 80%, but their learning stability would differ based on the maximum number of trials that were correct in a row (e.g., 40/40 correct trials were in a row, stability = 1; 20/40 correct trials were in a row, stability = 0.5). As a result, stability is independent from overall performance. In Experiment 1, there were no significant differences in stability across RB (*M* = 0.43) and II tasks (*M* = 0.44, *t*(85) = 1.09, *p* = 0.28, *d* = 0.12, 95% CI [−0.050, 0.015], Fig. 6b). Learning stability was unrelated across tasks (*r*(84) = −0.090, 95% CI [−0.30, 0.12], *p* = 0.41; Fig. 6c). The results directly replicated in Experiment 2 – there were no significant differences in stability across RB (*M* = 0.43) and II tasks (*M* = 0.41, *t*(92) = 0.98, *p* = 0.33, *d* = 0.10, 95% CI [−0.014, 0.043]; Fig. 6e) and learning stability was unrelated across tasks (*r*(91) = 0.061, 95% CI [−0.14, 0.26], *p* = 0.56; Fig. 6f).

In summary, during RB and II learning, participants did not significantly differ in accuracy, applied whichever strategy they used at no different levels of consistency, and did not differ in their speed to reach a median level of performance. These behaviors are consistent within an individual across different tasks, reflecting a stable trait or tendency. However, the same individual also tailored their strategy to the task and learning stability was unrelated across tasks. This may indicate that these behaviors are flexibly modulated by the task and/or by the learner’s state.

### Which behaviors support success in RB and II tasks?

**Fig. 8:**
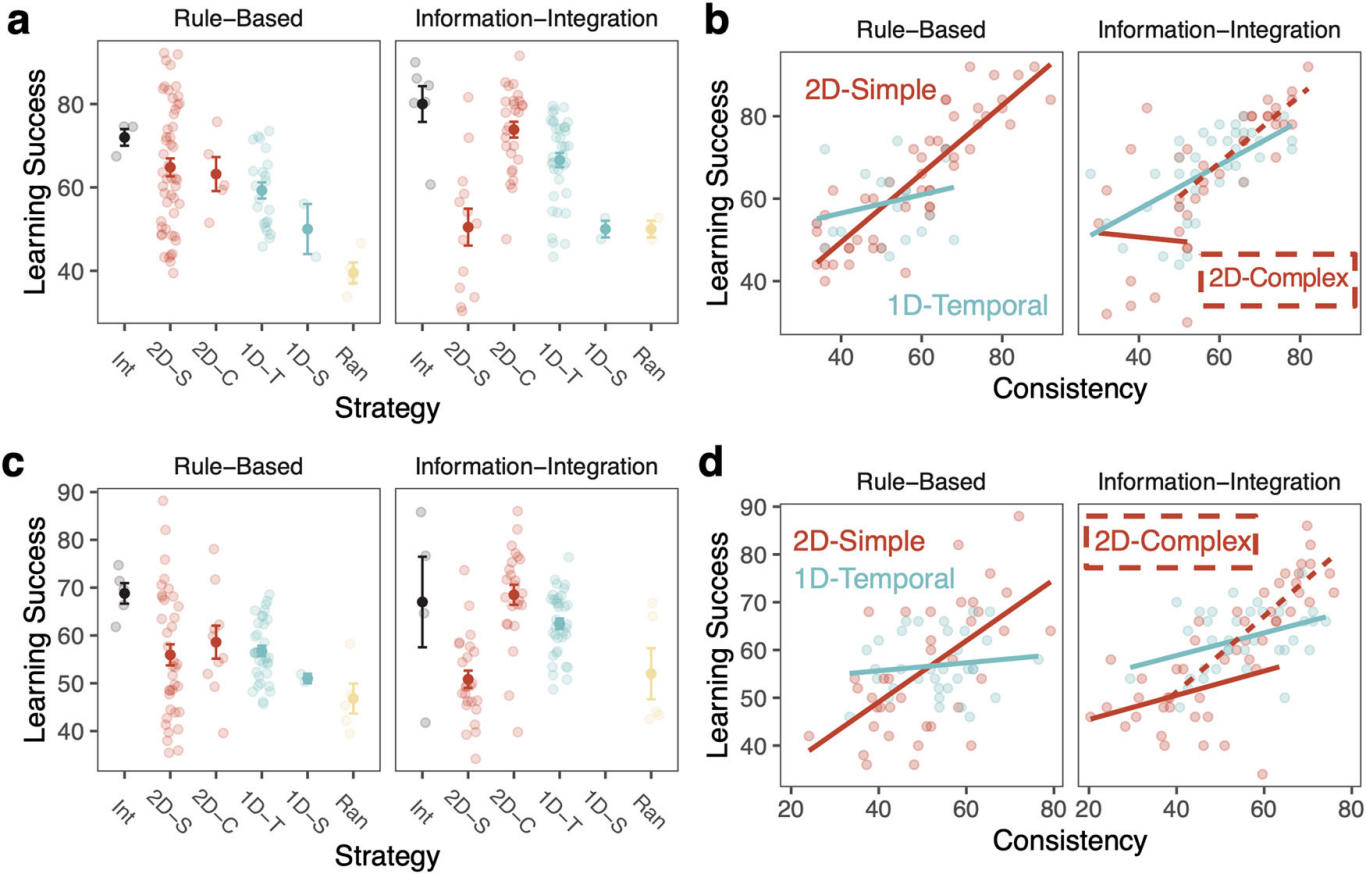
Relationship between learning strategies, strategy consistency, and success. For Experiment 1 (**a**, **b**) and Experiment 2 (**c**, **d**), **a**, **c** learning success based on final-block strategy for across the two tasks. Error bars reflect s.e.m. **b**, **d** Relationship between final-block strategy consistency and learning success based on the final-block learning strategy.

**Fig. 9:**
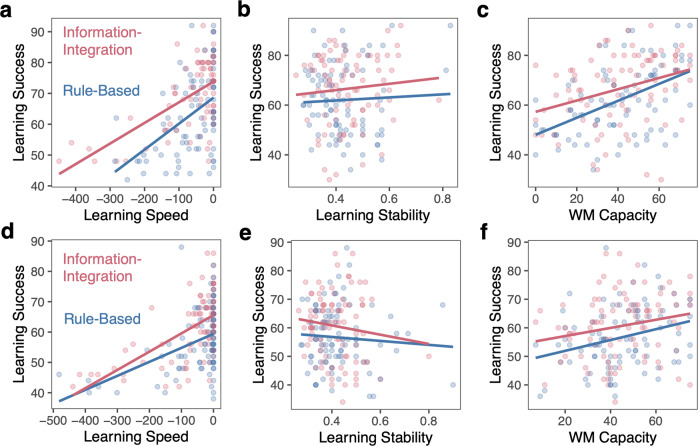
Relationship between learning speed, stability, and working memory and success. For Experiment 1 (**a**–**c**) and Experiment 2 (**d**–**f**), relationship between learning success and learning speed (**a**, **d**), learning stability (**b**, **e**), and working memory capacity (indexed by OSPAN score; **c**, **f**) across the RB and II tasks.

We next examine which behaviors support success in RB and II tasks and whether these are common or distinct across tasks. We examine whether common or distinct consistent strategies relate to performance across tasks (Fig. 8) and whether learning speed, stability, and individual differences in working memory capacity are related to learning outcomes in similar ways across tasks (Fig. 9).

#### Learning strategies and strategy consistency

Is using a particular type of strategy more beneficial for learning RB and II categories? As a reminder, the optimal strategy class for RB categories is 2D-Simple rules, whereas the optimal class for II categories is Integration. Importantly, even if participants are using an ‘optimal’ strategy, they may vary in how successfully they apply that strategy. Here, we ask if a particular type of strategy is associated with better learning success for RB and II categories (Fig. 7a, c). Because there were a mixed number of participants using different strategies, we focus on the strategies used by a substantial number of participants (RB: 2D-Simple and 1D-Temporal; II: 2D-Simple, 1D-Temporal, and 2D-Complex).

Across both experiments in the RB task, participants who used an optimal 2D-Simple strategy (Experiment 1: *M* = 65%, Experiment 2: *M* = 56%) did not have significantly different levels of success from those using a 1D-Temporal strategy (Experiment 1: *M* = 59%, *t*(63.6) = 1.91, *p* = 0.061, *d* = 0.44, 95% CI [−0.26, 11.3]; Experiment 2: *M* = 58%, *t*(61.9) = 0.68, *p* = 0.50, *d* = 0.16, 95% CI [−3.10, 6.32]). Even so, there was a wide range of success with either of these strategies and some participants found high levels of success with an optimal 2D-Simple strategy.

Across both experiments in the II task, there were significant differences in learning success based on learners’ strategies (Experiment 1: *F*(2, 73) = 18.8, *p* < 0.0001, *η*_G_^2^ = 0.34; Experiment 2: *F*(2, 81) = 9.50, *p* < 0.0001, *η*_G_^2^ = 0.19). According to Bonferroni-corrected post-hoc tests, participants who used 2D-Complex or 1D-Temporal strategies had significantly higher accuracy than participants who used 2D-Simple strategies (*p*s < 0.001; Experiment 2: *ps* < 0.012). In Experiment 1, participants who used 2D-Complex strategies also had significantly higher accuracy than participants who used 1D-Temporal strategies (*p* = 0.040), but these were not significantly different in Experiment 2 (*p* = 0.43).

In summary, different strategies are associated with learning success in the RB and II tasks. Specifically, in the RB task, participants’ accuracies were not significantly different when they used an optimal 2D-Simple strategy or a 1D-Temporal strategy. In the II task, participants were the most successful when they used either 2D-Complex or 1D-Temporal strategies. We note that very few participants across either experiment used an Integration strategy in either task, even though this strategy was optimal in the Integration task. We return to this point in the Discussion.

We next examined the relationship between strategy consistency and learning success for the RB and II tasks separately (because the tasks have different optimal strategies) using linear regression models (Fig. 7b, d). As before, we limited our analyses to strategies used by a substantial number of participants. In the RB task, the optimal 2D-Simple strategy was treated as the baseline and in the II task, the 2D-Complex strategy was treated as the baseline. In the RB task, the more consistently participants used a 2D-Simple strategy, the more successful they were (Experiment 1: *β*_consistency_ = 0.83, SE = 0.072, *p* < 0.0001; Experiment 2: *β*_consistency_ = 0.64, SE = 0.12, *p* < 0.0001). The relationship between consistency and success when using 1D-Temporal strategy was significantly weaker than when using a 2D-Simple strategy (Experiment 1: *β*_consistency*1D_ = −0.61, SE = 0.18, *p* < 0.0001; Experiment 2: *β*_consistency*1D_ = −0.56, SE = 0.20, *p* = 0.0075).

In the II task, the more consistently participants used a 2D-Complex strategy, the more successful they were (Experiment 1: *β*_consistency_ = 0.82, SE = 0.19, *p* < 0.0001; Experiment 2: *β*_consistency_ = 0.79, SE = 0.15, *p* < 0.0001). The more consistently participants used a 1D-Temporal strategy, the more successful they were; this relationship was not significantly different from 2D-Complex in Experiment 1 (*β*_consistency*1D-T_ = −0.29, SE = 0.23, *p* = 0.22), but was significantly weaker in Experiment 2 (*β*_consistency*1D-T_ = −0.56, SE = 0.18, *p* = 0.0033). In contrast to the RB task, consistency in using a 2D-Simple strategy was unrelated to success in the II task and this was significantly weaker than the 2D-Complex strategy in both Experiment 1 (*β*_consistency*2D-S_ = −0.92, SE = 0.38, *p* = 0.017) and Experiment 2 (*β*_consistency*2D-S_ = −0.54, SE = 0.19, *p* = 0.0064).

In all, these results demonstrate that the strategies participants use and the consistency of applying a particular strategy are distinctly related to success in the RB and II tasks. For RB tasks, the more consistently one applied a 2D-Simple strategy the more successful they were, and this relationship was stronger than for the 1D-Temporal strategy. For II tasks, the more consistently one can apply either a 1D-Temporal or 2D-Complex strategy (but not 2D-Simple), the more successful they were. Consistent application of the 2D-Complex strategy was most robustly associated with II learning success.

#### Learning speed, stability, and working memory capacity

Next, we examined how our measures of learning speed, stability, and general WM capacity were related to RB and II learning success using linear regression models.

For both RB and II tasks and in both experiments, learning speed was *positively* related to learning success (Fig. 9a, d; Experiment 1: *β*_speed_ = 0.084, SE = 0.016, *p* < 0.0001; Experiment 2: *β*_speed_ = 0.046, SE = 0.010, *p* < 0.0001). The faster one reached median level of performance, the better their ultimate learning accuracy. The relationship between learning speed and success was not significantly different across RB and II tasks (Experiment 1: *β*_speed*II_ = −0.017, SE = 0.020, *p* = 0.40; Experiment 2: *β*_speed*II_ = 0.014, SE = 0.014, *p* = 0.30). Quickly reaching some relatively achievable baseline level of performance gives participants ‘room to grow’ and continue to improve their accuracy, regardless of which type of category they were learning.

For both RB and II tasks and in both experiments, learning stability was *unrelated* to learning success (Fig. 9b, e; Experiment 1: *β*_stability_ = 6.12, SE = 14.7, *p* = 0.68; Experiment 2: *β*_stability_ = −6.93, SE = 10.1, *p* = 0.50) and was not significantly different across RB and II tasks (Experiment 1: *β*_stability*II_ = 6.52, SE = 21.1, *p* = 0.76; Experiment 2: *β*_stability*II_ = −9.27, SE = 16.8, *p* = 0.58). Having long streaks of correct responses, controlling for overall accuracy, was not associated with better learning in either task. This indicates that initial learning does not depend on when correct responses are made, only that correct responses are made.

For both RB and II categories and in both experiments, WM capacity was positively related to learning success (Fig. 9c, f; Experiment 1: *β*_WM_ = 0.34, SE = 0.069, *p* < 0.0001; Experiment 2: *β*_WM_ = 0.19, SE = 0.069, *p* = 0.0067) and was not significantly different across RB and II tasks (Experiment 1: *β*_WM*II_ = −0.11, SE = 0.098, *p* = 0.25; Experiment 2: *β*_WM*II_ = −0.047, SE = 0.098, *p* = 0.63). The higher one’s WM capacity, the better their ultimate learning accuracy, regardless of the type of category.

### Order effects

As these experiments used within-subjects designs with categories situated in the same stimulus space, we tested for carryover effects in both accuracy and strategies to understand what, if anything, participants brought from their first task experience to the second task. As a reminder, we attempted to control for carryover effects by having participants complete the tasks in two sessions separated by at least one week. In terms of accuracy, participants might either have higher accuracy in the second task based on general familiarity with the stimulus space or, alternatively, participants might have lower accuracy in the second task because they first needed to unlearn the categories they learned in the first session.

**Fig. 10:**
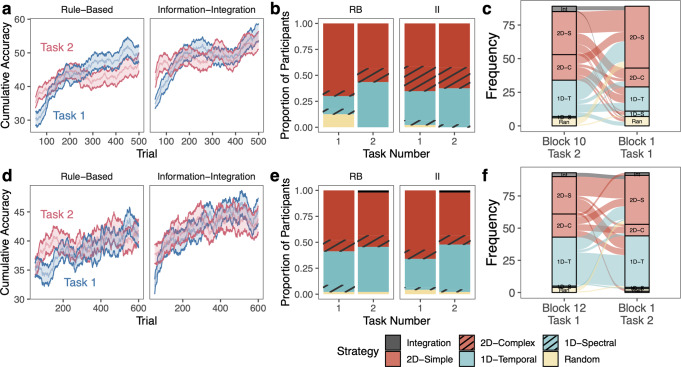
Order effects across experiments. For Experiment 1 (**a**–**c**) and Experiment 2 (**d**–**f**), **a**, **d** Cumulative accuracy based on the order the tasks were completed. Error ribbons reflect s.e.m. **b**, **e** Learning strategies in the two tasks based on the order the tasks were completed. **c**, **f** Learning strategies in the final block of the first task and the first block of the second task.

We compared average accuracy within the first 50 trials in both tasks based on whether participants completed the task first or second. In Experiment 1, there was an early slight benefit to performing a task second compared to first with an average accuracy increase of 4.33% in the RB task (*t*(81.5) = 1.89, *p* = 0.063, *d* = 0.41) and 6.43% in the II task (*t*(78.4) = 2.55, *p* = 0.013, *d* = 0.55). However, this effect was short-lived (Fig. 10a). After around 100 trials, there were no substantial differences in accuracy in the tasks regardless of the order in which participants completed the tasks. In Experiment 2, there were no significant differences in performance for the first task and the second task, with an average difference of 1.31% in the RB task (*t*(87.8) = 0.71, *p* = 0.48, *d* = 0.15) and 0.16% in the II task (*t*(86.9) = 0.078, *p* = 0.94, *d* = 0.016) with no clear difference across trials (Fig. 10d).

Next, to understand if there were any differences in strategies in the two tasks based on the order in which participants completed the tasks, we compared the strategies participants used in the first block of both tasks (Fig. 10b, e). In Experiment 1, the strategies participants used in the RB task significantly differed based on whether participants completed the task first or second (Fisher’s exact test, *p* = 0.00030) – when completing the RB task first, 65% of participants used an optimal 2D-Simple strategy, compared to 43% when completing the RB task second. When completing the RB task second, 43% used a 1D-Temporal strategy, compared to 13% who completed the RB task first. However, in Experiment 2, there were no significant differences in the strategies participants used in the RB task based on the task order (*p* = 0.45). For both experiments, the strategies participants used in the II task did not significantly differ based on task order (Experiment 1: *p* = 0.92; Experiment 2: *p* = 0.49). Because the differences did not replicate in Experiment 2, we believe that the differences in RB strategy based on task order in Experiment 1 may be an idiosyncratic effect of these participants, rather than reflecting true order effects.

Finally, to understand whether the strategies directly carried over across the tasks, we examined the strategy they used in the final block of the first task and first block of the second task (Fig. 10c, f). In Experiment 1, 53% of participants used a different general strategy (Integration, 2D, 1D, Random) and 67% of participants used a different specific strategy subclass (Integration, 2D-Simple, 2D-Complex, 1D-Temporal, 1D-Spectral, Random) across tasks. In Experiment 2, 43% used a different general strategy and 53% used a different strategy subclass. This pattern is highly similar to a control comparison of strategies in the first block of the first task and the final block of the second task (Experiment 1: 55% different general strategy, 72% different strategy subclass; Experiment 2: 56% different general strategy, 67% different strategy subclass). As a result, we do not believe that participants directly transferred their strategies from the first to the second task and, instead, tailored their strategies to the specific task.

In summary, in Experiment 1 we observed small, fleeting task order effects on accuracy even when separating sessions by over a week to minimize these effects. These effects were relatively small and disappeared around 100 trials in training and were not present in Experiment 2. While we found some differences in block 1 strategies depending on task order, this was limited to the RB tasks and did not replicate in Experiment 2. There was no clear carryover of strategies from the first task to the second task.

## Discussion

**Fig. 11:**
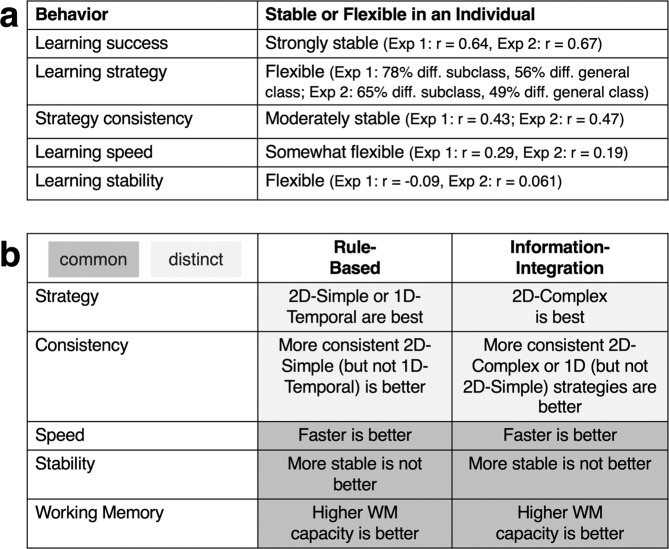
Summary of the individual behaviors and factors supporting RB and II learning. **a** Summary of how individual behaviors are stable or flexible across categories. **b** Summary of behaviors and abilities that are common or distinct in supporting RB and II category learning.

This study had three primary goals – (1) to describe a taxonomy of behaviors during category learning (Fig. 2), (2) to identify which behaviors are stable or flexible in the same individual across tasks, and (3) to identify whether these behaviors commonly or distinctly relate to learning success for RB and II categories. All together, we found evidence for both stable and flexible individual behavior in category learning (Fig. 11a) as well as both common and distinct factors supporting RB and II learning (Fig. 11b). We replicated the main results from Experiment 1 in Experiment 2 with different learners and categories with identical distributions.

Across perceptually similar auditory categories and procedurally identical tasks, some behaviors were stable within a learner across tasks (learning success, strategy consistency), whereas others were flexible across tasks (learning speed, learning strategies, learning stability). The primary focus of the literature on RB and II category learning has been to illustrate a dissociation (or lack thereof) between the types of categories at the group level^7,8,51^. The present investigation demonstrates that there are differences in how the same individual solves very similar category learning problems, highlighting the necessity for considering learning mechanisms at the level of an individual learner.

Regardless of how learners approached the learning tasks, overall learning outcomes were strongly correlated – better learners were better learners across different tasks. The ability to be successful across different kinds of tasks may relate to some task-independent stable trait abilities such as WM capacity. Supported by our own findings, individuals with higher WM capacity tend to have better learning outcomes across a wide array of tasks, including category learning^39,45,47,49,53,54,56^.

It is also possible that learning success is related to some internal, self-defined performance target that is specific to and stable for an individual learner. We did not inform participants about the highest possible accuracy in each of the tasks. While we assumed that their goal was to reach the highest possible accuracy, it is possible that each learner determined their own internal goal for performance level, monitored by their experienced ratio of correct to incorrect feedback. Once participants reached their internal goal, they may have been disincentivized or unmotivated to further improve their performance^76–78^. Some learners may be more motivated to push beyond their self-defined performance benchmark goals (explicit or implicit), whereas others may be content to maintain a certain level of performance^78^. This ability could be related to stable individual differences in in intrinsic motivation^79^ or need-for-cognition^80,81^, wherein participants might be intrinsically motivated to perform well on these tasks because the challenge itself is rewarding.

It is also possible that some learners may have believed that high levels of accuracy were impossible to reach in these difficult tasks. This belief may have led participants to understand the highest possible accuracy they could achieve in the second task as whatever level of performance they achieved in the first task. As a result, learners may have sought to reach that same accuracy in the second task, but not improve beyond that, regardless of how well they performed. This could be driven by a performance bias^82^ or by their own beliefs about their ability to perform in these tasks^83^. If instead learners were explicitly informed that near-perfect accuracy was possible or had been otherwise further incentivized to reach high levels of accuracy (e.g., with a performance-contingent bonus), this may have increased overall learning success. Future work could examine how participants’ beliefs about what is possible during a task (whether true or false) influence learning.

Regardless of the type of strategies participants used, how consistently they applied those strategies was moderately correlated across tasks. Strategy consistency may be related to an individual’s explore-exploit tendencies^84–86^. For example, someone who very consistently applies a strategy (regardless of how good of a strategy it is), may be exploiting known information. In contrast, someone who applies a strategy inconsistently may be exploring different kinds of strategies or specific criteria within the same class of strategy (e.g., placement of a decision boundary). As a result, when we define a strategy based on an entire block of responses (as we must do with current applications of strategies implemented here), we may be missing quick shifts in strategies that could reflect more exploratory strategy behavior. This kind of exploratory behavior may be good for learning over time compared to exploiting or consistently applying a suboptimal strategy.

Strategy consistency may also relate to lapses in attention or impoverished memory of one’s current strategy. That is, if participants are distracted on a given trial, they likely will not consistently apply the strategy they are holding in mind. Similarly, if participants forget what their in-mind strategy was, they may not apply the strategy consistently across trials. In future research, it would be informative to increase precision of the estimation strategies participants are using, how they switch between them at a more rapid timescale^38,87^, and how strategy consistency relates to explore/exploit tendencies across individuals. These questions may be particularly well-suited for online physiological measures (e.g., pupillometry) that may capture rapid processes that may be difficult to observe directly from behavior.

In contrast to learning success and strategy consistency, learning strategies were mostly unrelated across tasks – most people used different strategies during II and RB learning. It is important to note that RB and II categories had different optimal strategies, and this was explicitly part of the experimental design. Participants *should have* altered their strategies to perform well in the tasks. However, this was not an inevitability. Participants could have shown some biases to use strategies that could have carried over across tasks. Instead, learners flexibly changed their strategy to fit the demands of the task.

In another context, we may have expected more similarities in strategies across tasks. For example, in an experiment where learners learn RB categories in two different stimulus spaces (e.g., spectral-temporal ripples as examined here and nonspeech tones varying in pitch frequency/duration), it would have been much more likely that learners would use the exact same type of strategy (e.g., 2D-Simple rules) to learn the different tasks. Here, by examining tasks with different optimal strategies we were able to demonstrate that participants were not simply inclined to do what they did before in the other task and, instead, sought out new solutions to the new task problem in the same stimulus space.

The use of different strategies may have also been implicitly encouraged by the task instructions. At the beginning of the second session, participants were explicitly told that the upcoming task was different from the first task they completed: “Today, you will be doing a very similar task to what you did last time. You will learn four new categories of sounds using feedback. The sounds may sound similar to what you heard before, but the categories are different. Be sure to listen carefully to learn the new categories.” The explicit instruction and awareness that the categories were different across sessions may have encouraged participants to use different strategies across tasks. That is, this may have been an explicit cue to limit the influence of their learning in the first task because this knowledge would conflict with the current task. This is reminiscent of findings from problem solving where, unless otherwise instructed, participants may apply the same strategy from a prior problem, even when it is not useful for solving a second problem^88–90^. The ability to limit the influence of prior strategies reflects the ability relinquish prior knowledge that may not be helpful in the current task, which likely relates to cognitive flexibility^91^. Future work should elaborate on the effect of the specific relationship (i.e., align or conflict) between two tasks to understand how strategies are related across tasks.

We found that learning speed was somewhat flexible across tasks, with a weak correlation between learning speeds across tasks in Experiment 1 and no significant correlation in Experiment 2. We defined learning speed somewhat arbitrarily based on the number of trials it took to reach median performance for a given task. Many participants quickly reached this benchmark within the first 50 trials in at least one of the tasks. Several variables could determine how quickly a learner reaches this group baseline performance that could depend more on the task and less on the learner, leading to speed being flexible across tasks. For example, if a learner was more engaged with the task from the beginning, they could reach the baseline faster. If participants were encouraged to explore different kinds of strategies, they may reach the baseline slower but may be ultimately able to learn better^92^. Finally, it is possible that learners may have stumbled upon a strategy that initially helped them reach this baseline level of performance in a given task, but this discovery might be spontaneous and depend on the task, rather than some active strategy on behalf of the learner.

Finally, we found that learning stability was not significantly correlated across the two tasks, which could be related to the modest learning performance in these tasks. Overall, participants generally had very short streaks of correct responses in all tasks (Experiment 1: RB: *M* = 8.66 trials, II: *M* = 10.1 trials; Experiment 2: RB: *M* = 7.37 trials, II: *M* = 7.75 trials). While correct streaks, on average, increased over the course of learning, especially among those who performed well, streaks were still short compared to what would be expected if participants learned very well and had highly stable performance. If we examined learning when accuracy was very high, stability would likely be more reflective of lapses in attention (e.g., low stability reflects many attention lapses) with infrequent incorrect responses among long streaks of correct responses.

Additionally, it is likely that idiosyncrasies in stimuli could have affected the measure of learning stability. Stimuli were randomly selected across trials and differed in how difficult they were to categorize (i.e., distance from other categories). Even if someone had a high streak of correct responses, they may have broken the streak due to either an attentional lapse or because of random selection of a particularly difficult stimulus. These different sources of errors that may disrupt streaks of correct responses should be considered in future studies.

In summary, in initial learning, stability measures may be dependent on idiosyncrasies in the stimuli and accuracies may be too low to observe meaningful streaks. However, it is possible that learning stability may be stable in an individual learner across tasks at later stages of learning when overall performance is much higher and correct streaks are longer. At later stages of learning, stability could reflect lapses in attention or general distractibility^91^.

In the Introduction, we outlined separate possibilities for either mostly common or mostly distinct behaviors supporting RB and II learning. In the ‘mostly common’ view, common behaviors should support both RB and II learning because the underlying task is fundamentally the same (e.g., categorizing sounds in a supervised learning task; single system views of category learning). In contrast, in the ‘mostly distinct’ view, distinct behaviors should support RB and II learning due to differences in the fundamental mechanisms supporting learning (e.g., dual systems views of category learning). We found evidence of both common and distinct factors that supported RB and II category learning.

Faster learning speeds are better for learning across tasks. The underlying tasks for RB and II learning are the same – supervised category learning. The tasks only differed in how stimuli mapped onto categories. The faster learners could reach a baseline level of performance, the more room they had to grow and continue to learn, regardless of whether the task was RB or II. Faster learning speeds may reflect use of explicit-system rules^63,71^, which were very common here even in the II tasks. As such, our results suggest that initial learning gains (possibly within the entirety of our single sessions here) may be more likely to reflect explicit-system or hypothesis testing processes. The faster that learners could find a ‘good enough’ rough rule-based strategy, the more time they would have to refine and improve on this strategy to improve their performance. It is possible that the overwhelming use of explicit system strategies may also have been encouraged by the supervised nature of the learning tasks, with participants having a bias to find rules to separate the categories that can more easily be tested and refined by feedback. This would be in line with tendencies for adult learners to use rules during initial learning^14,93–95^.

Higher working memory capacity relates to better learning across tasks. Prior work has consistently demonstrated that higher working memory ability is associated with better RB category learning^39,45,54,73^, but the evidence for II learning is mixed^46–49^. Here, in the same individuals, same task, and same stimulus space, we found that learning success was positively correlated with WM ability for both RB and II tasks. Importantly, we also note that most participants used a rule-based kind of strategy during both kinds of tasks (e.g., 2D-Simple, 2D-Complex, 1D). Though we have demonstrated that WM ability does not differentiate performance in the two tasks, moderate levels of success were possible with rule-based strategies, proposed to rely on the WM-dependent explicit system. Our results do not rule out the existence of systems of category learning that rely and do not rely on WM ability, but demonstrate that even in II tasks, participants can leverage strategies that may rely on WM ability to a reasonable degree of success.

Learning stability is unrelated to early learning success in either task. Emphasizing our discussion above, it is possible that we are not able to see meaningful differences in learning stability within these single sessions of learning as participants did not learn well enough to maintain long streaks of correct responses to calculate learning stability. Learning stability does not relate to early learning success, but it may relate to later learning success when longer streaks are more likely. Future studies should examine longer training tasks or training with simpler distributions to understand how learning stability relates to performance across RB and II tasks.

Distinct (consistent) strategies are better for RB or II learning. Distinct across tasks, 2D-Simple or 1D-Temporal strategies were the best for RB learning, but 2D-Complex strategies were best for II learning. Further, the more consistently learners applied 2D-Simple strategies, the better it was for RB, but not II learning and the more consistently learners applied 1D-Temporal or 2D-Complex strategies, the better it was for II, but not RB learning.

An important distinction made in COVIS is that the RB and II categories differ in the systems that are optimal for learning, rather than what individuals actually do during learning. As such, one might argue that just because there are common behaviors supporting RB and II category learning in the current study does not refute the existence of multiple systems of learning. Instead, it could indicate that these common factors are present because people primarily used the explicit system during learning, evidenced by the overwhelming use of 2D and 1D strategies during RB and II learning. Another possibility is that many participants might not be encouraged to use optimal integration strategies in the II tasks because these strategies are difficult and effortful or take much more training and experience^96,97^. Instead, participants stick with ‘good enough’ strategies that allows them to reach a self-defined ‘acceptable’ level of performance while retaining cognitive resources they might rather be spending on something else. Future studies should examine learning behaviors from initial acquisition to very high levels of accuracy to fully understand how these behaviors might be reflective of similar or distinct processes in RB and II learning.

Several other behavioral patterns were present in the current study and were not anticipated by the frameworks outlined above. First, we observed a distinct bias for participants who used 1D rules to use them along the temporal modulation dimension. This could indicate that the underlying acoustic dimensions defining the categories were not equally salient or available, which likely affected learning outcomes^23,66,98,99^. Future work should examine behavior in tasks with equally separable dimensions to test the generalizability of this framework to other dimensions.

Next, while the main aims of this study were not focused on directly compare RB and II learning performance, as has been done extensively in prior literature, we observed that in terms of maximum cumulative accuracy, II performance was higher than RB performance. This finding is not unprecedented^23^, but does conflict with typical findings of category learning in the visual modality where RB performance is often at least initially higher than II performance^9,13,14,100^. We believe this pattern can be explained by the relative success of different strategies in the two tasks. While optimal performance could be achieved by using a 2D-Simple strategy in the RB task (97% or 100% accuracy in Experiments 1 and 2) or an Integration strategy in the II task (95% or 100% accuracy in Experiments 1 and 2), participants often used strategies that were suboptimal but could still yield better-than chance performance (Table 1). In particular, the most common strategy in the II task was a 2D-Complex strategy that could have yielded a highest possible accuracy of 89% or 76% in Experiments 1 and 2, respectively. Alternative suboptimal strategies in the RB task could have yielded a highest possible accuracy of 50% or 54% in Experiments 1 and 2, respectively. As a result, much of the feedback participants received about the II categories was aligned with a 2D-Complex type of strategy. This suboptimal strategy still did not allow them to achieve perfect performance, but participants were still able to perform quite well. Future work might focus on better disincentivizing participants from using suboptimal strategies to see whether participants can shift from moderately successful, suboptimal strategies to highly successful, optimal strategies.

In the process of defining how behaviors are stable in an individual or commonly related to learning across tasks, we defined a taxonomy of behaviors during category learning. In this way, we provide a framework that can examine the nuances of behavior moving beyond accuracy which cannot provide information about how participants learn^101^ and learning strategy which might not itself reflect processes accurately^61,62^. Further, we move beyond the examination of WM as a single individual difference factor in learning that has now been established to support category learning, likely across different types of categories^39,45,47,49,53,54,56^. We believe that future studies can benefit from using these measures of behavior to paint a more nuanced picture of behavior, with different predictions based on theoretical models of learning.

In all, our results demonstrate that even with very similar categories and identical training tasks completed over a week apart, we see differences in how an individual solves the problem and the factors that relate to success. These results illustrate a need to move beyond theoretical perspectives that rely primarily on group-level behavior and acknowledge the flexibility and complexity with which individuals approach a learning task.

## Methods

In Experiments 1 and 2, across two sessions separated by at least one week, participants learned nonspeech auditory rule-based (RB) and information-integration (II) categories. The order of the two tasks was counterbalanced across participants. As a part of a larger project, all participants in Experiment 1 also completed a Mandarin tonal speech category learning task at the very end of the experiment (i.e., after the artificial category learning tasks). As this task is unrelated to the predictions of the current study, we do not present or discuss results from this unrelated speech task.

### Participants

In both experiments, participants were recruited through Prolific (www.prolific.co) and the experiment was administered using the online Gorilla Experiment Builder^102^. To assess individual differences in learning across different tasks, we aimed to collect a large sample and arbitrarily settled on recruitment of 100 participants in the first session in both experiments. Our final included sample size was 86 participants in Experiment 1 and 93 participants in Experiment 2. A post-hoc power analysis was run using the *pwr* package in R^103^ and indicated that a correlation of *r* = 0.34 between performance measures across tasks could be detected with a sample of 86 participants with statistical power at a .90 level with an alpha of .05. In Experiment 1, 100 participants ages 18-35 (45 F, *M* = 25.3 years, SD = 5.05 years) completed one session and 90 returned for a second session and 86 completed all tasks (36 F, *M* = 25.4 years, SD = 5.04 years). In Experiment 2, 99 participants ages 18–37 (42 F, *M* = 30.0 years, SD = 4.68 years) completed one session and 93 returned for a second session and completed all tasks (38 F, *M* = 30.1 years, SD = 4.66 years). The analyses included only participants who completed both RB and II tasks. Participants completed a language and music history questionnaire prior to participating. Participants provided written informed consent and received $10/hour for their participation for a total of $20 across two sessions. The study protocol was approved by the Institutional Review Board at the University of Pittsburgh.

### Stimuli

Stimuli for the RB and II tasks were artificial nonspeech ripples varying in temporal modulation and spectral modulation (Fig. 1). These dimensions are thought to be fundamental properties of complex sounds, including speech^104^ and have been posed as auditory analogs to visual Gabor patches^105^. These dimensions have also been used in prior investigations of auditory category learning^2,44,106,107^. Using nonspeech dimensions enables creation of complex, controlled artificial categories, as is common practice in research on artificial visual category learning (e.g., Gabor patches; line length and orientation). In Experiment 1, the category distributions were created by sampling from a bivariate normal distribution and had 300 total stimuli (75 stimuli/category; Table 2). Each category was sampled separately and then centered in a particular area of the two-dimensional space to form the RB and II categories. To control for potential biases induced by the inherent structure of the categories, in Experiment 2, the underlying structures of the RB and II categories are identical. The entire category space was sampled from a bivariate normal distribution and had 300 total stimuli (75 stimuli/category). Participants in Experiment 2 therefore encountered the same stimuli in both tasks. The only difference between RB and II categories was the mapping of stimulus region to category label.

**Table 2:** Category distribution information.

Category	Category means (temporal Hz, spectral cyc/oct)	Variance (temporal Hz, spectral cyc/oct)	Covariance
Experiment 1 – Rule-based			
Category 1	6.18, 0.57	1.43, 0.091	0.036
Category 2	6.18, 1.77	1.43, 0.090	0.033
Category 3	11.0, 0.57	1.43, 0.090	0.031
Category 4	11.0, 1.77	1.43, 0.090	0.033
Experiment 1 – Information-integration			
Category 1	4.60, 0.96	1.54, 0.087	0.037
Category 2	8.08, 1.79	1.54, 0.087	0.032
Category 3	8.08, 0.14	1.54, 0.087	0.033
Category 4	11.55, 0.96	1.54, 0.087	0.032
Experiment 2 – Rule-based			
Category 1	5.41, 0.86	3.88, 0.11	0.0048
Category 2	5.33, 1.83	3.17, 0.12	0.090
Category 3	11.0, 0.73	3.54, 0.13	0.15
Category 4	11.3, 1.86	3.81, 0.11	0.050
Experiment 2 – Information-integration			
Category 1	4.32, 1.34	2.51, 0.15	0.059
Category 2	8.29, 2.07	6.07, 0.076	0.036
Category 3	8.35, 0.62	4.60, 0.10	−0.093
Category 4	12.4, 1.38	2.44, 0.18	−0.0016

In both experiments, the RB categories can be optimally learned with a conjunctive rule that is orthogonal to the component dimensions (Fig. 1a; e.g., high/low on spectral modulation and high/low on temporal modulation). The II categories require rules that are non-orthogonal to the component dimensions, are less clearly verbalizable and include joint consideration of the two dimensions (Fig. 1a; i.e., diagonal bounds). Stimuli were 1s in duration and RMS amplitude matched to 70 dB.

### Procedure

Participants completed two sessions, separated by approximately one week (Experiment 1: *M* = 8.40 days, SD = 1.29 days, range: 7.74–13.9 days; Experiment 2: *M* = 8.92 days, SD = 2.26 days, range: 7.87–22.0 days). In session 1, participants learned one type of category (RB or II) followed by an operation span task^108^ (OSPAN) as a measure of WM capacity. In session 2, participants completed the other task. In Experiment 1, participants also completed a Mandarin tone learning task, which was always completed last and is not discussed further as part of the current study. At the start of both sessions, participants completed a sound check to ensure that they could hear the sounds and that they were wearing headphones^109^.

### Category learning

In both tasks, participants were instructed to categorize the sounds into four equally likely categories and use feedback to be as accurate as possible. Participants learned the categories across ten 50-trial blocks. Stimuli were selected randomly without replacement and were presented dichotically for a duration of 1 sec. Participants made an untimed categorization response (1, 2, 3, or 4) and received feedback (“Correct”/“Incorrect”) for 1 sec followed by a 1 s ITI.

#### Working memory

To understand how differences in WM capacity might relate to learning outcomes, participants completed an operation span (OSPAN) task^108^. The OSPAN task was chosen as a measure of working memory because it can reliably measure WM capacity and relates to other measures of relevant abilities such as attention and complex cognitive abilities^110^.

Participants were shown simple arithmetic problems, reported whether the presented solutions were correct or incorrect (e.g., (9 + 5) × 1 = 14), and were then shown a letter on the screen (e.g., W). Participants saw 15 letter sequences that spanned from three to seven letters. After a full sequence was presented, participants recalled the letters presented in order. The OSPAN score was calculated as the sum of the length of all the correctly recalled spans. For instance, if a participant correctly recalled the sequence of four letters, four points were added to their score. We did not filter the scores based on accuracy of the arithmetic problems as it does not change the validity of working memory span tasks^111^.

#### Learning strategies

To assess participants’ strategies during learning, we applied several classes of decision bound computational models^58,60,112^ as well as a striatal pattern classifier (SPC) model and random responder model. Decision bound models divide the two-dimensional space into categories with decision boundaries that are proposed to rely on implicit or explicit learning processes^112^. We fit several classes of models that make different assumptions: an implicit SPC/Integration model, explicit two-dimensional (2D) and one-dimensional (1D) rule models, and a random responder model. For 1D and 2D rule models, the general procedure was that the model takes a set of a participant’s responses to the stimuli across a set of trials (e.g., 50 trials) and finds the boundaries in stimulus space that best account for that participant’s pattern of responses. For the SPC model, the model finds locations of pseudo-prototypes in the stimulus space that best account for the participants’ pattern of responses. The input of the models is the trial-wise response data and dimensional information about the stimuli (i.e., *x*- and *y*-dimension coordinates, Fig. 1).

The implicit Striatal Pattern Classifier model is a neurobiologically grounded model thought to reflect procedural learning mechanisms^112^. The Integration model assumes that participants use feedback to learn stimulus-response associations instantiated within the striatum^113^ and can be thought of as complex version of an exemplar model^10^. This model has nine free parameters: eight that determine the location of hypothetical striatal units in perceptual space and one that represents the noise associated with the placement of the units. When a participant uses an Integration strategy, they combine information across both dimensions in a manner that is optimal for II categories (confirmed by running models on the II stimulus distributions).

**Fig. 12:**
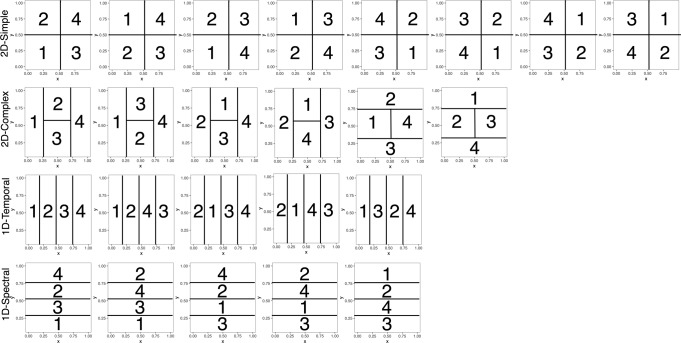
Model Versions. Versions of models fit with different category response mappings.

The second class of models represents explicit, hypothesis-testing mechanisms and includes 2D and 1D rule models. Simple 2D rule (2D-Simple) models place two decision boundaries (one along each dimension) that are combined to determine category membership. When a participant uses a 2D-Simple rule strategy, they selectively attend to both dimensions. This type of strategy is optimal for the RB categories (confirmed by running models on the RB stimulus distributions). The 2D-Simple rule model has three free parameters: two for the boundaries along the x- and y-dimensions and one noise parameter. We also fit a complex 2D (2D-Complex) rule model that places three decision boundaries (two alone one dimension and one along the other dimension). This model is also referred to as a ‘conjunctive-H’ model as the boundaries take the shape of an H in the stimulus space, distinguishing from high/low values on a primary dimension with two decision boundaries, and intermediate values on the primary dimension with the decision boundary along the other dimension. That is, for 2D-Complex rule models, two categories can be identified by one dimension alone, while the other two categories sit at intermediate values of the primary dimension and must use the second dimension to be differentiated. In contrast, 1D rule models assume that the participant sets three decision boundaries along only one of the dimensions (Temporal or Spectral) and have four free parameters: three for boundaries along the relevant dimension and one noise parameter. We fit several versions of the 1D and 2D rule models that assume different mappings of categories onto regions of the stimulus space (Fig. 12).

Finally, the random responder model assumes that the participant guesses on each trial.

The models were run in Python, version 3.7.4^114^. The model parameters were estimated using maximum likelihood procedures^115^ and model selection used the Bayesian Information Criterion (BIC), which penalizes models with more free parameters: BIC = r*ln*N* - 2ln*L*, where *r* is the number of free parameters, *N* is the number of trials in a given block for a given subject, and *L* is the likelihood of the model given the data^116^. In Experiment 1, models were fit to each of the ten 50-trial blocks for each participant in both tasks (10 blocks × 2 tasks × 86 participants = 1720 total best-fit models) and in Experiment 2, models were fit to each of the 12 50-trial blocks for each participant in both tasks (12 blocks × 2 tasks × 93 participants = 2232 total best-fit models). Within each block for each participant, the model with the lowest BIC value was selected as the best-fitting model.

#### Assessing model accuracy

We assessed the ability of the best-fit models to account for participant response patterns by comparing the best-fit model’s predicted response to the participant’s actual response. This enabled an assessment of model accuracy. Overall, the models captured participants’ response patterns better than chance (95% cumulative probability with *P* success = 0.25 and *n* = 50, chance-level performance = 25 ± 9%; Experiment 1: RB *M* accuracy: 52%; II *M:* 55%; Experiment 2: RB *M* accuracy: 50%; II *M:* 52%). Note that we expect these models to be below 100% accuracy because of lapses in attention or inconsistency of a participant to apply the same strategy in the same way over the entire course of 50 trials. The above-chance performance here indicates that the best-fit models can reliably detect patterns in participants’ responses that reflect these underlying strategies.

#### Model recovery simulations

We also assessed the ability of our models to capture relevant patterns in the data by simulating response data (so we can identify the true strategy) and applying the models in the same way as the human response data. For each of the possible response strategies (1D-Temporal, 1D-Spectral, 2D-Simple, 2D-Complex [2 versions], Integration, Random), we simulated response data 10 times (total of 140 simulated datasets, 70 for each task). For all trials, we applied a deterministic response strategy based on the model being simulated with parameters sampled randomly in reasonable ranges based on category distributions. For example, for the 1D-Temporal strategy, we simulated a strategy where stimuli with temporal modulation values less than or equal to 5 Hz, the response category was 1, ≤8.2 Hz, the response category was 2, ≤12 Hz, the response category was 3, and >12 Hz, the response category was 4.

Using this simulation approach, we analyzed the data in several ways to ensure model recovery. First, we determined the best-fit model compared to the simulated model. If the modeling approach as a whole is able to accurately determine a participants’ response strategy, then we should see high alignment between the ground truth strategy (determined by simulations) and the best-fit strategy (determined by models). Indeed, we found that 93% of Experiment 1 II, 97% of Experiment 1 RB, 94% of Experiment 2 II, and 97% Experiment 2 RB simulated datasets were best-fit by the ground truth strategy.

Next, we assessed the model’s prediction accuracy in similar ways to the human responses (predicted category response was compared to actual category response). The models accurately captured the simulated responses with an average accuracy of 97% for the Experiment 1 II, 99% for the Experiment 1 RB, 95% for the Experiment 2 II, and 98% for the Experiment 2 RB simulated datasets.

Finally, we assessed whether the best-fit model fit parameters aligned with the simulated parameters (i.e., for 1D/2D rule models: boundaries; for SPC/integration model: placement of the striatal units; for Random model: probability of responses for each category choice). As evidence of good fit, the models accurately estimated the ground truth parameters of the estimated data for both Experiment 1 (*r* = 0.96) and Experiment 2 (*r* = 0.98). In Experiment 1, there were two outliers for the II task and Integration strategy (same simulated subject) where the fit values for one of the hypothetical striatal units (unit 1) was far from the true values. This seemed to be due to this model responding “category 1” only once out of the 300 total stimuli based on the randomly sampled parameters, which is uncommon for human participants.

The data were analyzed using R, version 4.2.1^117^, with the R packages *tidyverse*, version 1.3.2^118^ and *rstatix*, version 0.7.0^119^. Computational models were run using custom scripts in python. Data visualizations were created using the R packages *ggplot2*, version 3.3.5^120^, *ggalluvial*, version 0.12.3^121^, and *ggthemes*, version 4.2.0^122^.

### Inclusion and ethics

We have complied with all relevant ethical regulations. The study protocols were approved by the Institutional Review Board at the University of Pittsburgh. We obtained informed consent from all participants. All participants who completed the experimental tasks were included in analyses.

### Reporting summary

Further information on research design is available in the Nature Portfolio Reporting Summary linked to this article.

## Supplementary information

Supplemental material

Reporting summary

## Data Availability

All data and stimulus materials (including for model recovery analyses) are publicly available at the Open Science Framework and can be accessed at 10.17605/OSF.IO/TFB6A^123^.

## Code availability

Code for generating stimuli, conducting analyses, and visualizing data can be requested directly from Casey L. Roark. The experiment and task designs can be accessed through Gorilla’s Open Materials (https://app.gorilla.sc/openmaterials/556657).
