# Supplementary material for: Stable, flexible, common, and distinct behaviors support rule-based and information-integration category learning
Source: NPJ Sci Learn. 2023 May 13;8:14. doi: 10.1038/s41539-023-00163-0 (PMC10183008; doi:10.1038/s41539-023-00163-0)
Supplement: Supplementary file 1 — Supplemental material [file 41539_2023_163_MOESM1_ESM.pdf]

Supplementary Information

To address potential concerns about using the same response data to fit the strategies and assess strategy consistency, we conducted supplementary analyses splitting the trials used in the two analyses. Odd-numbered trials were used for fitting the strategies and even-numbered trials were used for assessing strategy consistency. Due to the reduced number of trials, we combined blocks to run strategies and assess learning consistency (i.e., blocks 1/2, 3/4, 5/6, 7/8, 9/10, 11/12). This allowed for each 'block' of trials to have 50 trials to obtain an estimation of strategy over a reasonable number of trials (e.g., Wasserman, 2010; vs. 25 trials in each block). Because we collapsed across blocks, this approach removes some ability to examine changes of strategies over time (relative to all blocks) and introduces a broader assumption that strategies are stable across multiple blocks (e.g., over 100 trials rather than 50 trials). We ran these supplementary analyses on the Experiment 2 data (same distributions between RB and II categories, 600 trials in each task, 93 participants).

The results of these supplementary analyses assessing strategies and strategy consistency using different sets of trials were well-aligned with our analyses estimating strategies and consistency across all trials. Just as in our primary analyses, we examined patterns of learning strategy across blocks (here, groups of two blocks: Supplementary figure 1a) and in the final block across tasks (here, final two blocks: Supplementary figure 1b). We also assessed the consistency of learning strategy across blocks (Supplementary figure 1c) and the relationship of final two block strategy consistency across tasks (Supplementary figure 1d).

Overall, we found that the most common strategies were 2D-Simple and 1D-Temporal for RB and 1D-Temporal for II. Relative to fitting the models with all trials in 50-trial blocks, there were slightly more participants best-fit by 1D-Temporal in both RB and II tasks and more 2D-Complex strategies in RB tasks with odd-numbered trials. Just as with all trials, strategies were generally unrelated across tasks (66% different specific strategy, 48% different general strategy). Strategy consistency assessed with even-numbered trials across the final group of trials (i.e., final two blocks) was not significantly different across RB and II tasks ($t(85) = 1.42$, $p = .16$, $d = 0.15$, 95% CI [-0.80, 4.86]), but consistency was moderately correlated across tasks ($r(84) = .46$, p < .001). These results are identical to our results when strategy and strategy consistency were both assessed with all trials.

We also found similar results regarding how strategies (Supplementary figure 2a) and strategy consistency (Supplementary figure 2b) were associated with learning success in the RB and II tasks. In line with our results with all trials, in the RB task, accuracies did not significantly differ based on the strategies participants used ($F(2, 78) = 1.83$, $p = .17$, $\eta_G^2 = .045$; 2D-Simple: M = 57%, 2D-Complex: M = 53%, 1D-Temporal: M = 59%). In the II task, accuracies differed across strategy types ($F(2, 83) = 19.8$, $p < .001$, $\eta_G^2 = 0.32$). According to Bonferroni-corrected post-hoc tests and exactly in line with our results with all trials, participants using either 2D-Complex (M = 66%) or 1D-Temporal (M = 62%) strategies had significantly higher accuracy than those using 2D-Simple strategies (M = 49%, ps < .001), but were not significantly different from one another ($p = .14$).

Supplementary figure 1

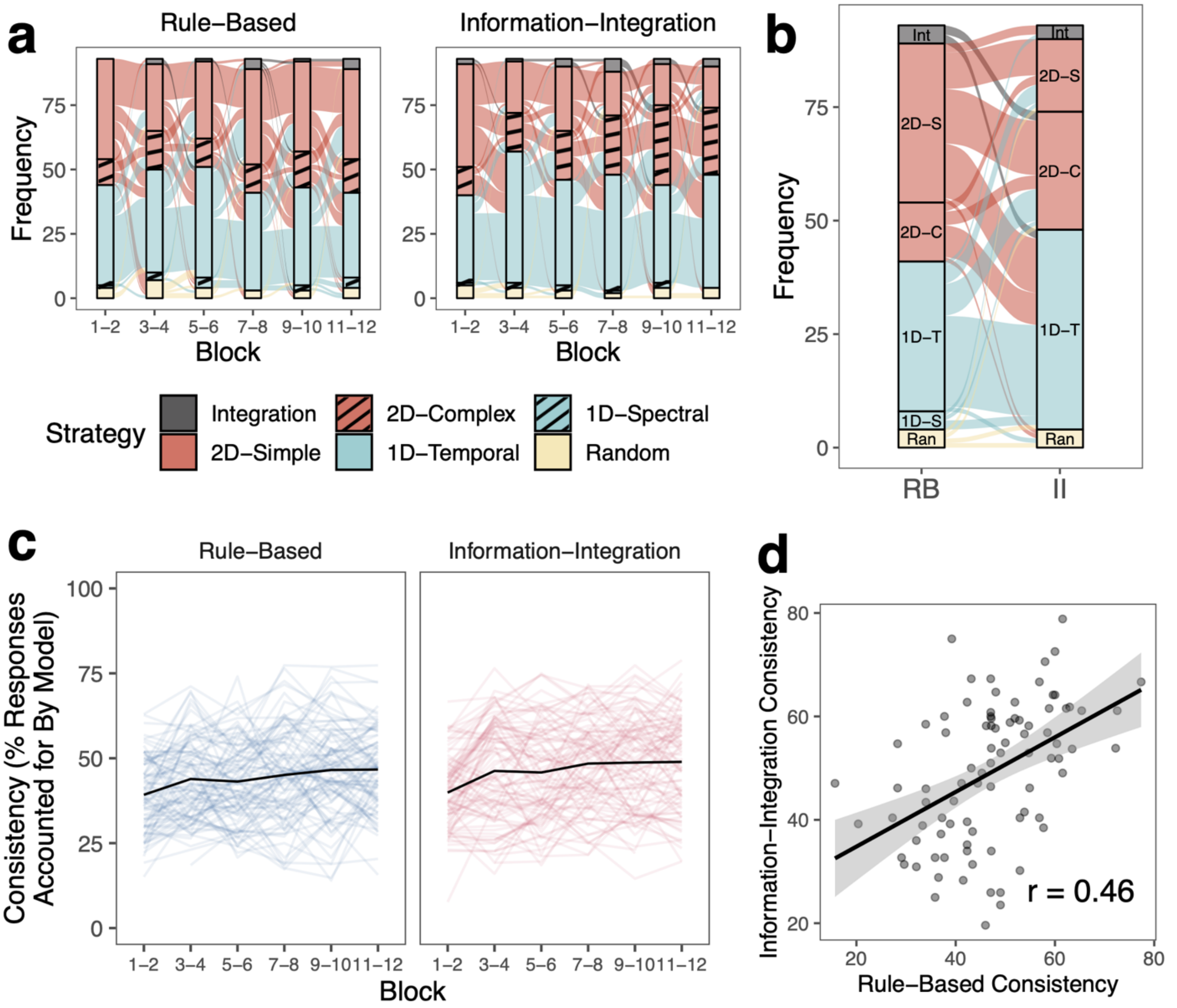

Note. For Experiment 2 (a) Strategies across blocks of 50 trials, assessed using odd-numbered trials. Colors reflect different classes of strategies, which are binned by frequency. Connections between blocks show how strategies changed across blocks. (b) Alluvial plot showing the strategies the same participants used in the final block of the RB and II tasks. (c) Consistency of strategy application measured as the percent of a participant's responses on even-numbered trials that were accounted for by the best-fitting model/strategy fit using odd-numbered trials. 100% consistency would reflect that the participant clearly applied this strategy with no exceptions in any trials. 25% consistency would reflect a relatively poor fit of the model to the participant's left-out data. Consistency is only measured for non-random strategies. Mean is shown as the black line and individual data is shown in colored lines. (d) Correlation between final two-block consistency in the RB and II tasks. Error ribbon reflects *SEM.*

In examining how different levels of strategy consistency were associated with learning success across tasks, we found that for RB categories, the more consistently learners applied a 2D-

Simple strategy, the better their learning success ($\beta_{consistency} = 0.68$, SE = 0.11, $p < .001$). This relationship was significantly weaker for the 1D-Temporal strategy ($\beta_{consistency*1D\text{-}T} = -0.44$, SE = 0.17, $p = .011$) but was not significantly different for the 2D-Complex strategy ($\beta_{consistency*2D\text{-}C} = -0.14$, SE = 0.29, $p = .62$. For II categories, the more consistently learners applied a 2D-Complex strategy, the better their learning success ($\beta_{consistency} = 0.70$, SE = 0.10, $p < .001$. This relationship was significantly weaker for both 1D-Temporal ($\beta_{consistency*1D\text{-}T} = -0.41$, SE = 0.14, $p = .0039$) and 2D-Simple strategies ($\beta_{consistency*2D\text{-}S} = -0.69$, SE = 0.20, $p = .0010$. These results are identical to our findings with all trials.

To summarize, whether we run the strategies in separate 50 trial blocks with all trials or odd-numbered trials only and whether we assess strategy consistency with all trials or even-numbered trials only, our findings are nearly identical. There are some differences in the number of participants best-fit by different strategies, which is somewhat expected because the odd-numbered trials cover a larger span of trials (100 vs. 50 trials). Even so, our patterns of the relationship between strategies and strategy consistency across RB and II tasks as well as how strategies and strategy consistency are associated with accuracy in the final block(s) have identical patterns whether we use all trials or separate sets of trials to assess strategy and strategy consistency.

Supplementary figure 2

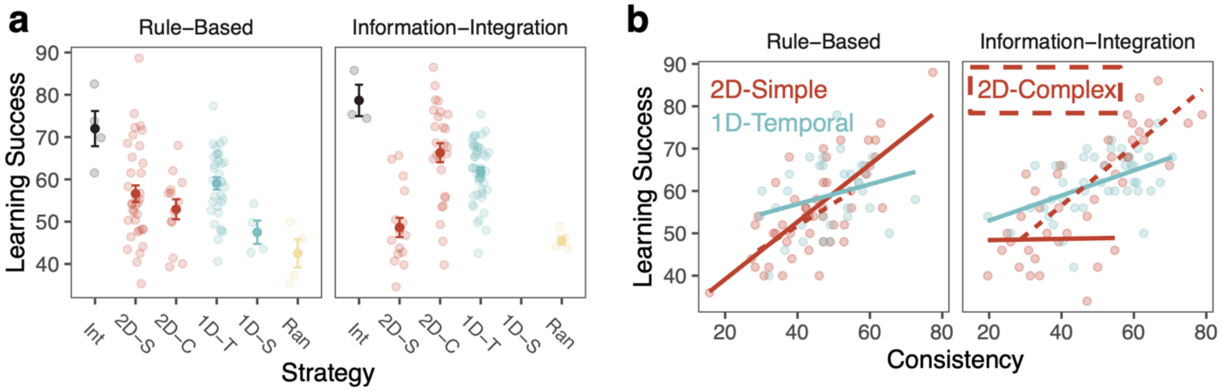

Note. For Experiment 2, (a) learning success based on final two-block strategy assessed with odd-numbered trials for across the two tasks. Error bars reflect *SEM.* (b) Relationship between final two-block strategy consistency (based on even-numbered trials) and learning success based on the final-block learning strategy (based on odd-numbered trials). Note that we only included conditions for which there were a substantial number of participants using that strategy.
