# Supplementary material for: Stable, flexible, common, and distinct behaviors support rule-based and information-integration category learning
Source: NPJ Sci Learn. 2023 May 13;8:14. doi: 10.1038/s41539-023-00163-0 (PMC10183008; doi:10.1038/s41539-023-00163-0)
Supplement: Supplementary file 2 — Reporting summary [file 41539_2023_163_MOESM2_ESM.pdf]

nature portfolio

Corresponding author(s): Casey L. Roark, Bharath Chandrasekaran

Last updated by author(s): 02/14/2023

Reporting Summary

Nature Portfolio wishes to improve the reproducibility of the work that we publish. This form provides structure for consistency and transparency in reporting. For further information on Nature Portfolio policies, see our Editorial Policies and the Editorial Policy Checklist.

Statistics

For all statistical analyses, confirm that the following items are present in the figure legend, table legend, main text, or Methods section.

n/a	Confirmed	
☐	☒	The exact sample size (*n*) for each experimental group/condition, given as a discrete number and unit of measurement
☐	☒	A statement on whether measurements were taken from distinct samples or whether the same sample was measured repeatedly
☐	☒	The statistical test(s) used AND whether they are one- or two-sided<br>*Only common tests should be described solely by name; describe more complex techniques in the Methods section.*
☐	☒	A description of all covariates tested
☐	☒	A description of any assumptions or corrections, such as tests of normality and adjustment for multiple comparisons
☐	☒	A full description of the statistical parameters including central tendency (e.g. means) or other basic estimates (e.g. regression coefficient) AND variation (e.g. standard deviation) or associated estimates of uncertainty (e.g. confidence intervals)
☐	☒	For null hypothesis testing, the test statistic (e.g. *F*, *t*, *r*) with confidence intervals, effect sizes, degrees of freedom and *P* value noted<br>*Give P values as exact values whenever suitable.*
☒	☐	For Bayesian analysis, information on the choice of priors and Markov chain Monte Carlo settings
☒	☐	For hierarchical and complex designs, identification of the appropriate level for tests and full reporting of outcomes
☐	☒	Estimates of effect sizes (e.g. Cohen's *d*, Pearson's *r*), indicating how they were calculated

Our web collection on statistics for biologists contains articles on many of the points above.

Software and code

Policy information about availability of computer code

Data collection	Data collection was completed using Gorilla Experiment Builder, which is a freely available website for research design and data collection. Code for experiments is directly available: https://app.gorilla.sc/openmaterials/556657
Data analysis	The data were analyzed using R, version 4.2.182, with the R packages tidyverse, version 1.3.283 and rstatix, version 0.7.084. Computational models were run using custom scripts in python. Data visualizations were created using the R packages ggplot2, version 3.3.585, ggalluvial, version 0.12.386, ggthemes, version 4.2.087, and phonTools, version 0.2-2.188.

For manuscripts utilizing custom algorithms or software that are central to the research but not yet described in published literature, software must be made available to editors and reviewers. We strongly encourage code deposition in a community repository (e.g. GitHub). See the Nature Portfolio guidelines for submitting code & software for further information.

Data

Policy information about availability of data

All manuscripts must include a data availability statement. This statement should provide the following information, where applicable:

- Accession codes, unique identifiers, or web links for publicly available datasets
- A description of any restrictions on data availability
- For clinical datasets or third party data, please ensure that the statement adheres to our policy

All data and stimulus materials are publicly available at the Open Science Framework and can be accessed at https://doi.org/10.17605/OSF.IO/TFB6A.

Human research participants

Policy information about studies involving human research participants and Sex and Gender in Research.

Reporting on sex and gender	Experiment 1: One hundred participants ages 18-35 (45 F, M = 25.3 years, SD = 5.05 years) completed one session and 90 returned for a second session and 86 completed all tasks (36 F, M = 25.4 years, SD = 5.04 years). In Experiment 2, 99 participants ages 18-37 (42 F, M = 30.0 years, SD = 4.68 years) completed one session and 93 returned for a second session and completed all tasks (38 F, M = 30.1 years, SD = 4.66 years).<br><br>We identified this as self-reported sex information. We did not run analyses on the basis of sex.
Population characteristics	Experiment 1: One hundred participants ages 18-35 (45 F, M = 25.3 years, SD = 5.05 years) completed one session and 90 returned for a second session and 86 completed all tasks (36 F, M = 25.4 years, SD = 5.04 years).). In Experiment 2, 99 participants ages 18-37 (42 F, M = 30.0 years, SD = 4.68 years) completed one session and 93 returned for a second session and completed all tasks (38 F, M = 30.1 years, SD = 4.66 years).
Recruitment	Participants were recruited through Prolific (www.prolific.co). We restricted participation based on prior participation in studies with similar stimuli and recruited participants aged 18-37 who reported being English speaking monolinguals.
Ethics oversight	The study protocol was approved by the Institutional Review Board at the University of Pittsburgh.

Note that full information on the approval of the study protocol must also be provided in the manuscript.

Field-specific reporting

Please select the one below that is the best fit for your research. If you are not sure, read the appropriate sections before making your selection.

☐ Life sciences ☒ Behavioural & social sciences ☐ Ecological, evolutionary & environmental sciences

For a reference copy of the document with all sections, see nature.com/documents/nr-reporting-summary-flat.pdf

Behavioural & social sciences study design

All studies must disclose on these points even when the disclosure is negative.

Study description	This study is a quantitative experiment of category learning.
Research sample	The research sample involved young adults (ages 18-37) who were recruited through the Prolific online recruitment study. Experiment 1: One hundred participants ages 18-35 (45 F, M = 25.3 years, SD = 5.05 years) completed one session and 90 returned for a second session and 86 completed all tasks (36 F, M = 25.4 years, SD = 5.04 years).). In Experiment 2, 99 participants ages 18-37 (42 F, M = 30.0 years, SD = 4.68 years) completed one session and 93 returned for a second session and completed all tasks (38 F, M = 30.1 years, SD = 4.66 years). This study sample was chosen to ensure minimal influence of exposure to multiple languages, which affects category learning ability. This is a standard, non-representative sample.
Sampling strategy	The sampling procedure was a convenience sample based on participation on the Prolific recruitment website. A post-hoc power analysis was run using the pwr package in R104 and indicated that a correlation of r = 0.34 between performance measures across tasks could be detected with a sample of 86 participants with statistical power at a .90 level with an alpha of .05. Our sample sizes of 86 and 93 across experiments ensure that reasonable effects can be observed if they exist.
Data collection	We collected data using the online Gorilla Experiment Builder (gorilla.sc; Anwyl-Irvine et al., 2019). Participants completed the study on their own computers and the research did not directly interact with participants during data collection.
Timing	Experiment 1 data was collected between 2020-08-31 AND 2020-09-14. Experiment 2 was run in response to reviewer comments and data was collected between 2023-01-03 and 2023-01-25.
Data exclusions	No data were excluded from the analyses.
Non-participation	Across sessions, 14 participants failed to complete or return to complete Experiment 1 and 6 participants failed to complete or return to complete Experiment 2.
Randomization	All participants completed all tasks, with the order of category learning tasks randomly counterbalanced across participants.

Reporting for specific materials, systems and methods

We require information from authors about some types of materials, experimental systems and methods used in many studies. Here, indicate whether each material, system or method listed is relevant to your study. If you are not sure if a list item applies to your research, read the appropriate section before selecting a response.

Materials & experimental systems

n/a	Involved in the study
☒	☐ Antibodies
☒	☐ Eukaryotic cell lines
☒	☐ Palaeontology and archaeology
☒	☐ Animals and other organisms
☒	☐ Clinical data
☒	☐ Dual use research of concern

Methods

n/a	Involved in the study
☒	☐ ChIP-seq
☒	☐ Flow cytometry
☒	☐ MRI-based neuroimaging
